# The developments of cyan emitting phosphors to fulfill the cyan emission gap of white-LEDs

**DOI:** 10.3389/fchem.2023.1274410

**Published:** 2023-10-17

**Authors:** Noor Zamin Khan, Sayed Ali Khan, Weilong Chen, Muhammad Amin Padhiar, Muhammad Tahir Abbas, Zakir Ullah, Marcin Runowski, Xin Xu, Ren-Kui Zheng

**Affiliations:** ^1^ School of Physics and Material Sciences, Guangzhou University, Guangzhou, China; ^2^ Hoffmann Institute of Advanced Materials, Shenzhen Polytechnic, Shenzhen, China; ^3^ Department of Chemistry and Chemical Biology, Rutgers University, Piscataway, NJ, United States; ^4^ Hefei National Laboratory for Physical Sciences at Microscale and Department of Physics, University of Science and Technology of China, Hefei, China; ^5^ Beijing Key Laboratory of Multiphase Flow and Heat Transfer for Low Grade Energy Utilization, North China Electric Power University, Beijing, China; ^6^ Departamento de Física, Universidad de La Laguna, Santa Cruz de Tenerife, Spain; ^7^ Faculty of Chemistry, Adam Mickiewicz University, Poznań, Poland; ^8^ CAS Key Laboratory of Materials for Energy Conversion, School of Chemistry and Materials Science, University of Science and Technology of China, Hefei, China

**Keywords:** full-visible-spectrum, garnet-type compound, cyan-emitting, warm-white LEDs, stability

## Abstract

Future generations of solid-state lighting (SSL) will prioritize the development of innovative luminescent materials with superior characteristics. The phosphors converted into white light-emitting diodes (white LEDs) often have a blue-green cavity. Cyan-emitting phosphor fills the spectral gap and produces “full-visible-spectrum lighting.” Full-visible spectrum lighting is beneficial for several purposes, such as light therapy, plant growth, and promoting an active and healthy lifestyle. The design of cyan garnet-type phosphors, like Ca_2_LuHf_2_Al_3_O_12_ (CLHAO), has recently been the subject of interest. This review study reports a useful cyan-emitting phosphor based on CLHAO composition with a garnet structure to have a cyan-to-green emitting color with good energy transfer. It could be employed as cyan filler in warm-white LED manufacturing. Due to its stability, ability to dope with various ions suitable for their desired qualities, and ease of synthesis, this garnet-like compound is a great host material for rare-earth ions. The development of CLHAO cyan-emitting phosphors has exceptionally high luminescence, resulting in high CRI and warm-white LEDs, making them a viable desire for LED manufacturing. The development of CLHAO cyan-emitting phosphors with diverse synthesis techniques, along with their properties and applications in white LEDs, are extensively covered in this review paper.

## 1 Introduction

White LEDs are the most used practical white light source since they can be used in various technologies and offer the most significant advantages, including high luminous efficiency, reliability, and chemical stability. The combination of host lattice and activators (Host: activator) known phosphors plays an essential role in developing optically driven white LEDs (Duke et al., 2018; Guo and Huang, 2018; Huang and Guo, 2018; Hakeem et al., 2019; Zhang Q. et al., 2021; Li et al., 2022; Xu et al., 2022). The combination of yellow-emitting Y_3_Al_5_O_12_: Ce^3+^ (YAG: Ce^3+^) phosphors and blue-emitting (440–480 nm) InGaN chips are currently used in commercial w-LEDs for solid-state lighting. The white LEDs obtained because of this combination generate poor-quality white light due to excellent light generation because of the deficiency of the red color component. The poor color quality limits the use of this combination for general illumination (Geng et al., 2018; Wu et al., 2019). Another technique involves covering a UV-emitting chip with phosphors emitting red, green, and blue light. This combination produces white light with high color quality. However, the cyan emission color gap means white light is still behind ideal. The quality of light produced by white LEDs in artificial lighting is significantly determined by the lighting efficacy (LE), color rendering index (CRI), and correlated color temperature (CCT)

The phrase refers to evaluating a light source’s ability to faithfully replicate the colors of diverse objects compared to a perfect reference light (such as incandescent or natural light). The CRI values range from 0 to 100. The color quality of the white light produced will be lower when the CRI is lower. Similarly, CRI values over 80 are typically required for general lighting. There is a substantial demand for high-CRI (Ra>90) light sources in numerous fields, including photography, movies, museums, and art galleries. The emission spectrum of a light source must be broad enough to achieve a high CRI value. “Full-visible spectrum illumination” has been suggested as a fresh idea for solid-state white lighting (Zhang et al., 2016; Huang, 2019a; Huang, 2019b; Yuan et al., 2019; Khan et al., 2021; Cao et al., 2022). It aims to generate a light source that matches natural sunlight regarding CRI and color temperature. It is challenging to depict colors adequately since there is still a cyan gap between blue and green emissions, peaking at 470 and 500 nm. The general illumination obtained because of the approaches mentioned above is likewise not appropriate in this region. To provide white light of the highest quality, novel cyan-emitting phosphors must be developed in this spectral range. To boost the optical performance of phosphor-converted LEDs (Pc-LEDs), the emission spectra of the devices are modified using a narrow-band, cyan-emitting phosphor with a slight Stokes shift or a broadband phosphor covering both the cyan and green spectral region (Wang et al., 2016; Strobel et al., 2018; Ding et al., 2021; Li et al., 2022; Wu et al., 2022). The narrow-band cyan-emitting phosphors compensate for the peak valley between blue and green emissions, which is crucial for boosting color reproduction.

Designing very stable UV/blue excitable cyan-producing phosphors is essential to attain a full visible light spectrum. An efficient cyan emission with a peak at 495 nm and a full width at half maximum (FWHM) of 32 nm has recently been reported for a narrow band BaSi_2_O_2_N_2_:Eu^2+^ phosphor (Wu et al., 2022). However, its layered crystal structure makes it chemically and thermally unstable. Another oxonitridoberylate phosphor was registered with a narrow-band emission peaking at 495 nm and a full width at half maximum (FWHM) of 35 nm for Sr[Be_6_ON_4_]: Eu^2+^ (Strobel et al., 2018). However, the phosphor’s toxicity and harsh synthetic conditions pose severe challenges to its implementation. It is required to create novel stable, non-toxic phosphors having narrow-band cyan emission to enhance the color rendering of pc-LEDs.

Another family of phosphor compositions is A_3_B_2_C_3_O_12_, which has different cation feasibility for A, B, and C ions and garnet type structure with different surrounding environments for Ce^3+^ and Eu^2+^ doped ion occupation. Because these dopants (Ce^3+^ and Eu^2+^) exhibit a 5d - 4f spin-permitted transition with tunable emission throughout the visible range, the Ce^3+^ or Eu^2+^ ions are frequently used as activators in various phosphors. Furthermore, this type of composition has high stability against moisture and heat. More interestingly, the Ce^3+^-activated garnet phosphors may exhibit much lower near-UV and blue light absorption, making them suitable for use with the most commercially available near-UV and blue excitable white LEDs. For example, the BaLu_2_Al_4_SiO_12_:Ce^3+^ garnet phosphors emit green emission peaking at 513 nm under 450 nm blue light excitation (Qiang et al., 2018).

This review article thoroughly discussed the development of various rare-earth ion-activated A_3_B_2_C_3_O_12_ garnet phosphors that emit highly efficient narrow-band cyan emission under UV and blue light excitation. First, we shall discuss the formation of various compositions by substituting various cations and their effects on the structure and luminescence features. Next, we shall shed light on cyan and green emission formation with co-doping of Ce^3+^/Tb^3+^ ions in the garnet-type structure to develop a single phase with wide-range tunable emission in the cyan and green spectral region. These phosphors’ composition in white LED fabrication was also discussed for practical applications in solid-state lighting and display application.

## 2 Crystal structure of garnet phosphors

Jaffe studied the role of the yttrium ions in garnet crystals and foresaw the double substitution of Y^3+^-Al^3+^ for Mn^2+^-Si^4+^ in a Mn_3_Al_2_Si_3_O_12_ garnet in 1951 (Jaffe, 1951). Yoder *et al.* also proposed the development of [Mn_1-x_Y_x_]_3_[Al]_2_[Si_1-x_Al_x_]_3_O_12_ solid solutions in the same year. This resulted in the final composition of Y_3_[Al]_2_[Al]_3_O_12_, which has a garnet-like structure (Yoder and Keith, 1951). In today’s world, it is common knowledge that in addition to the naturally occurring silicate minerals, there are other fabricated garnets with compositions including aluminate, gallate, and germanates.

**FIGURE 1:**
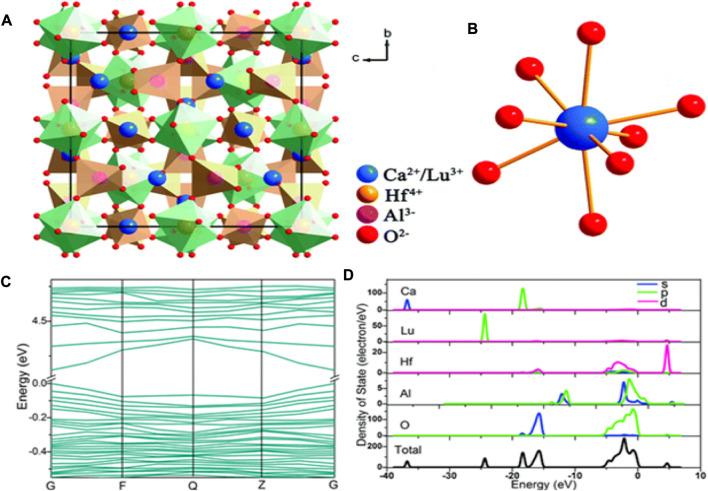
**(A)** Crystal structure of CLHAO (viewed along the а axis) and **(B)** coordination of Ca^2+^/Lu^3+^ **(C)** A diagram showing the CLHAO band structure. **(D)** (DOS) total and partial (Wang et al., 2016). Copyright 2016, Royal Society of Chemistry.

The coordinated Ca^2+^/Lu^3+^ ions and crystal structure of Ca_2_LuHf_2_Al_3_O_12_ (CLHAO) are shown in Figures 1A, B. One can see the occupation of Ca^2+^/Lu^3+^, Hf^4+^, and Al^3+^ in Wyckoff sites 24c, 16a, and 24d. There was strong octahedral and tetrahedral coordination between Hf^4+^ and Al^3+^, in which the octahedrons [HfO_6_] and the tetrahedrons [AlO_4_] were joined by O^2-^ ions. As a result, eight O^2-^ anions were coordinated with the Ca^2+^/Lu^3+^ cations, yielding a [(Ca/Lu)–O_8_] dodecahedron. This compound may exhibit good thermal stability because of the structure’s compactness and rigidity. Figure 1C depicts the band structure of Ca_2_LuHf_2_Al_3_O_12_ calculated from the refined crystal structure. The CLHAO compound was found to have a direct bandgap of 4.15 eV at the G point of the Brillouin zone, located between the maximum valence band and the minimum conduction band. The calculations show that CLHAO is an appropriate host material because it offers enough band gaps to occupy Ce^3+^ to serve as emission centers. As a result of the absence of phonons in the transition, the direct band gap is also more likely to be advantageous to luminescence than the indirect band gap (Kireev and Samokhvalov, 1978).

Figure 1D shows the total and partial densities of states (DOS and PDOS) of the developed CLHAO compound. Anti-bonding orbitals of the Hf-3d and O-2p states dominated the conduction band. The orbital O-2p contributes the most to the valence band out of all the atoms. The O-2p states were almost filled in the valence band, but the Ca-s and Lu-p states were less concentrated. This suggests that the Ca/Lu-O bond was ionic. The wide band of Hf, Al, and O states, which corresponds to the hard polyhedron of [HfO_6_] and [AlO_4_], indicates the covalent connection between the Hf-O and Al-O bonds (Zhao et al., 2016).

Zhang and others developed Ce^3+^-doped garnet phosphors that emit cyan under 400 nm (near-UV) light irradiation (Zhang Z. J. et al., 2021). The Ce^3+^ ions were doped in the 0.01, 0.02, 0.03, 0.06, and 0.08 ranges. The phase purity and crystal structure of the developed CLHAO:*x*Ce^3+^ phosphor was confirmed by x-ray diffraction (XRD). The surface morphology of the synthesized materials was checked with field-emission scanning electron microscopy (FE-SEM). Under near-UV (400 nm) light irradiation, a broadband emission in the cyan spectral region (477–493 nm) is highly efficient.

Interestingly, the thermal stability of the synthesized CLHAO:xCe^3+^ phosphors was excellent. To find the potential of the synthesized phosphors in the generation of white LEDs, a prototype of white LEDs was developed with a CRI ranging from 83.2 to 89.4.

**FIGURE 2:**
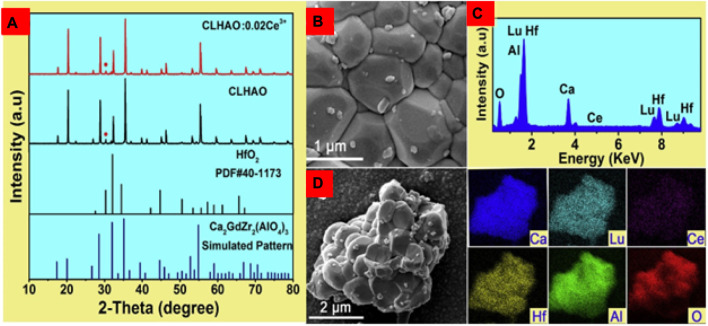
**(A)** An XRD characterization of the CLHAO:0.02Ce^3+^ phosphors and the host CLHAO. Card for Ca_2_GdZr_2_(AlO_4_)_3_ (COD ID-4338781), **(B)** A FE-SEM image, **(C)** EDS spectrum, and **(D)** Element mapping images of CLHAO:0.02Ce^3+^ (Zhang Z. J. et al., 2021). Copyright 2021, Elsevier.

The XRD pattern of the undoped CLHAO and Ce^3+^-doped CLHAO (CLHAO:0.02Ce^3+^) phosphors is illustrated in Figure 2A. These two samples show identical peak positions to Ca_2_GdZr_2_Al_3_O_12_ (COD ID-4338781). There is an impurity peak of HfO_2_ at around 30.36°. All the remaining peaks match well with those in the standard PDF card number. This result meant that the phases of samples are independent of the Ce^3+^ doping. The minute amount of the HfO_2_ impurity phase should not impact the optical characterization (Fischer et al., 2018). A FE-SEM image was used to examine the prepared phosphors. Figure 2B is an FE-SEM representation of the CLHAO as it was produced with a 0.02Ce^3+^ concentration, demonstrating the particles’ size range from 0.3 to 1.2 μm. Figure 2C shows the EDS spectra of CLHAO:0.02Ce^3+^ garnet phosphors. The distribution of the six components Ca, Lu, Al, Hf, and Ce on the produced phosphor was uniform, as shown in Figure 2D from the elemental mapping of CLHAO:0.02Ce^3+^ phosphors. As a result, CLHAO:Ce^3+^ phosphors were successfully prepared.

## 3 Photoluminescence properties

### 3.1 Occupation of Ce^3+^ in multiple crystallographic sites

The trivalent Ce^3+^ ion is considered one of the promising activators for phosphors converted to white–LEDs due to 5d—4f spin-allowed transitions. More specifically, the Ce^3+^ activated phosphors have a broadband absorption ranging from UV to near-UV and blue spectral region matching well to almost all available UV and blue emitting chips. Similarly, the emission of Ce^3+^ doped phosphors is broadly tunable in the entire spectral region. Furthermore, the 5d—4f transition of Ce^3+^ ions exhibit substantial variations in optical properties due to the strong crystal field of the host lattice.

The Ce^3+^ activated Ca_2_MZr_2_Al_3_O_12_ (M = Gd^3+^, La^3+^, and Lu^3+^) (Gong et al., 2014; Wang and Wang, 2015; Sun L. L. et al., 2020) and Ca_2_MHf_2_Al_3_O_12_: Ce^3+^ (M = Y^3+^, Gd^3+^, La^3+^, Lu^3+^) (Liang et al., 2020a; Liang et al., 2020b; Sun Q. et al., 2020; Zhang Z. J. et al., 2021) phosphors with the garnet structure emit an efficient narrow-band cyan emission, as reported recently. However, these phosphors show a stability issue with decreased luminescence intensity with increasing temperature. The intensity largely decreases when the temperature reaches 150°C (the operational temperature of the LED device) (Liang et al., 2020a). For example, the luminescence intensity of Ca_2_LaHf_2_Al_3_O_12_:Ce^3+^ phosphor decreases to 46.5% at 150°C, restricting its application in full-visible-spectrum LEDs (Liang et al., 2020b). Thermal stability can be improved by increasing the band gap of the materials (You et al., 2021), the structural stiffness (Brgoch et al., 2013; Denault et al., 2015; Zhuo et al., 2018), the incorporation of nitrides into the lattice (Park et al., 2013; Kim et al., 2016; Ding et al., 2021), the coating of phosphors with ceramic layers (such as SiO_2_ and TiO_2_) (Lee and Yoo, 2011; Zhuang et al., 2011; Pasinski et al., 2016; Zhu et al., 2018), and the development of solid solution phosphors (Lin et al., 2017; Li et al., 2021).

**FIGURE 3:**
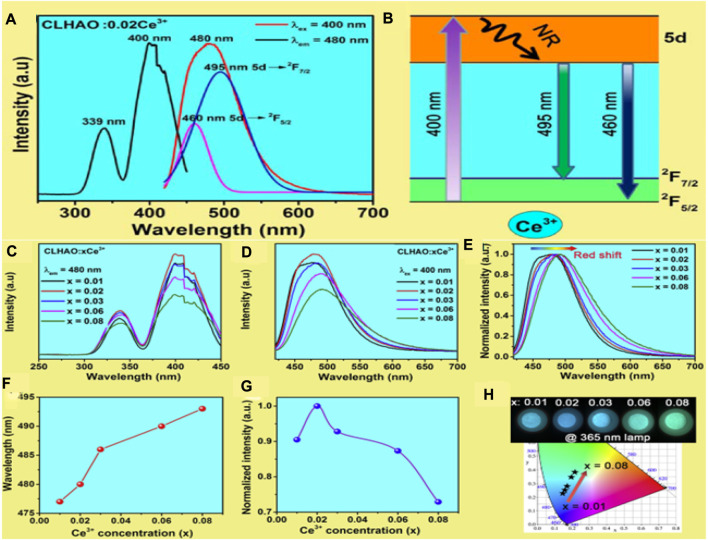
**(A)** PLE and PL spectra for the wavelength ranges 250–450 nm and 420–700 nm, respectively, for the material at room temperature with *x* = 0.02. **(B)** Ce^3+^ phosphors exhibit the Ce^3+^ ion energy levels by demonstrating the luminescence process at 400 nm excitation and **(C)** PLE spectrum, **(D)** PL spectrum, and **(E)** The *x*Ce^3+^ phosphors in the normalized PL spectrum of CLHAO (*x* = 0.01, 0.02, 0.03, 0.06, and 0.08). **(F)** In CLHAO:*x*Ce^3+^ phosphors excited at 400 nm; the PL peak position was correlated with the Ce^3+^ concentration. **(G)** In CLHAO:*x*Ce^3+^ phosphors, the integrated PL intensity normalized. **(H)** CIE of CLHAO:*x*Ce^3+^ phosphors (λ_ex_ = 400 nm). These phosphor samples are shown in the insets photographed with a 365 nm UV lamp (Zhang Z. J. et al., 2021). Copyright 2021, Elsevier.

Figure 3A depicts the PL and PLE spectra of their optimized CLHAO:0.02Ce^3+^ phosphors. The excitation (PLE) is broadband in the 300–450 nm range, peaking at 339 and 400 nm. The dominant peak was observed at 400 nm, attributed to 4f to 5d absorption. Under 400 nm near-UV light irradiation, the CLHAO:0.02Ce^3+^ phosphors show a broadband emission peak at 480 nm associated with a spin-allowed 5d to 4f transition. It was observed that the emission band is a dual-band emission under Gaussian fitting, leading to a difference of 1537 cm^-1^ attributed to the 5d → ^2^F_5/2_ and ^2^F_7/2_ transitions of Ce^3+^ dopants. Figure 3B demonstrates the Ce^3+^ ion energy level system in CLHAO:0.02Ce^3+^ phosphors. The electrons of the Ce^3+^ ion go into the 5d excited state by absorbing the excitation energy at wavelengths below 400 nm and close to UV stimulation. After that, a non-radiative mechanism leads the excited electrons to relax to the lowest energy level of the 5d excited state. When electrons go through the lowest exciting levels of type 5d and then transition back to their ground states of types 4f (^2^F_5/2_ and ^2^F_7/2_), they emit bright emissions in blue and cyan spectral regions.

The PLE spectra of the CLHAO:*x*Ce^3+^ phosphors (*x* = 0.01, 0.02, 0.03, 0.06, and 0.08) are shown in Figure 3C. The PL spectra with all the Ce^3+^ concentrations are broadband in the 300–450 nm range, with a maximum peak at 400 nm. The higher intensity was observed at *x* = 0.02. Similarly, the PL (Figures 3D, E) is a single broadband spectrum with red shifting with increasing Ce^3+^ concentration. The redshift appeared to be attributed to the shifting of Ce^3+^ ions to lower 5d levels with higher crystal field splitting. Tetrahedral, octahedral, and dodecahedral distortions in garnet structures are associated with competition between neighboring polyhedra, and the polyhedron’s size significantly impacts Ce^3+^ crystal-field splitting. There is a decrease in the octahedral interplanar distance when Ce^3+^ concentrations are high. The reason for PL peaks occupying longer wavelengths is due to an increase in crystal field splitting and lattice distortion. The shifting trend of these emission spectra with FWHM values is shown in Figure 3F. In the CLHAO:*x*Ce^3+^ phosphors, the wavelength gradually increases from 86 nm (at *x* = 0.01) to 97 nm (at *x* = 0.08), which is similar to other garnet-structured phosphors like (Gd_1-*x*_Lu_*x*_)Al_5_O_12_:Ce^3+^ (FWHM = 100–116 nm) and YAG:Ce^3+^ (FWHM = 106 nm) (Li et al., 2016). Because high levels of Ce^3+^ are added to CLHAO:*x*Ce^3+^ phosphors, their FWHM values may be higher because of this uneven broadening. Figure 3G shows the normalized integral PL intensity of the CLHAO:*x*Ce^3+^ phosphors in the 420–700 nm (ex = 400 nm). The luminescence of the CLHAO:*x*Ce^3+^ samples continuously rises as the Ce^3+^ ion doping concentration increases from *x* = 0.01 to *x* = 0.08 (Zhou et al., 2016). A concentration quenching effect can result in a decrease in luminescence intensity when *x* exceeds 0.02. The CIE chromaticity diagram for *x*Ce^3+^ phosphors stimulated at 400 nm is shown in Figure 3H. The digital photographs were taken under a 365 nm UV light lamp. The CIE chromaticity coordinates move from (0.152, 0.226) to (0.251, 0.379), indicating changes in the emission colors of *x*Ce^3+^ phosphors with increasing Ce^3+^ concentrations.

### 3.2 Doping of Cr^3+^ in multiple crystallographic sites

Phosphors with garnet structures have attracted much interest recently due to their excellent chemical and thermal stability. Garnet phosphors have the structural formula A_3_X_2_C_3_O_12_, where the A site coordinates with eight O ions, the X site coordinates with six O ions, and the C site coordinates with four O ions (Zheng et al., 2019). By substituting a simple composition, weak crystal strengths can be created for Cr^3+^ due to the abundance of coordination environments. The emission spectrum of Cr^3+^ ions tuning with various compositions due to different crystal fields associated with different compositions (Liu et al., 2015; Katayama et al., 2016; Skruodiene et al., 2016; Xu et al., 2017; Malysa et al., 2018; Zhang et al., 2018; Skruodiene et al., 2019; Wu et al., 2021). To understand the persistent luminescence of Cr^3+^ dopants, Katayama et al. used the electron trapping theory to detect a strong emission in YAG:Cr^3+^ at 690 nm from a ^2^E→ ^4^A_2_ transition (Katayama et al., 2016). The substitution of large cations leads to switching Cr^3+^ from dodecahedral to tetrahedral sites, according to Xu *et al.*, which brings efficient tuning in the emission spectrum in a wide spectral region (Xu et al., 2017). Similarly, the emission of X_3_Sc_2_Ga_3_O_12_:Cr^3+^ (X = Lu^3+^, Y^3+^, Gd^3+^, and La^3+^) phosphor is tuned with various cations at the X site (Malysa et al., 2018). More interestingly, the full nitride CaSiN_2_ phosphor has a weak crystal field compared to Ca_2_LuHf_2_Al_3_O_12_, which results in comparatively high thermal stability. Furthermore, it was observed that the developed Ca_2_LuHf_2_Al_3_O_12_:Cr^3+^ garnet phosphors could generate a broadband emission.

**FIGURE 4:**
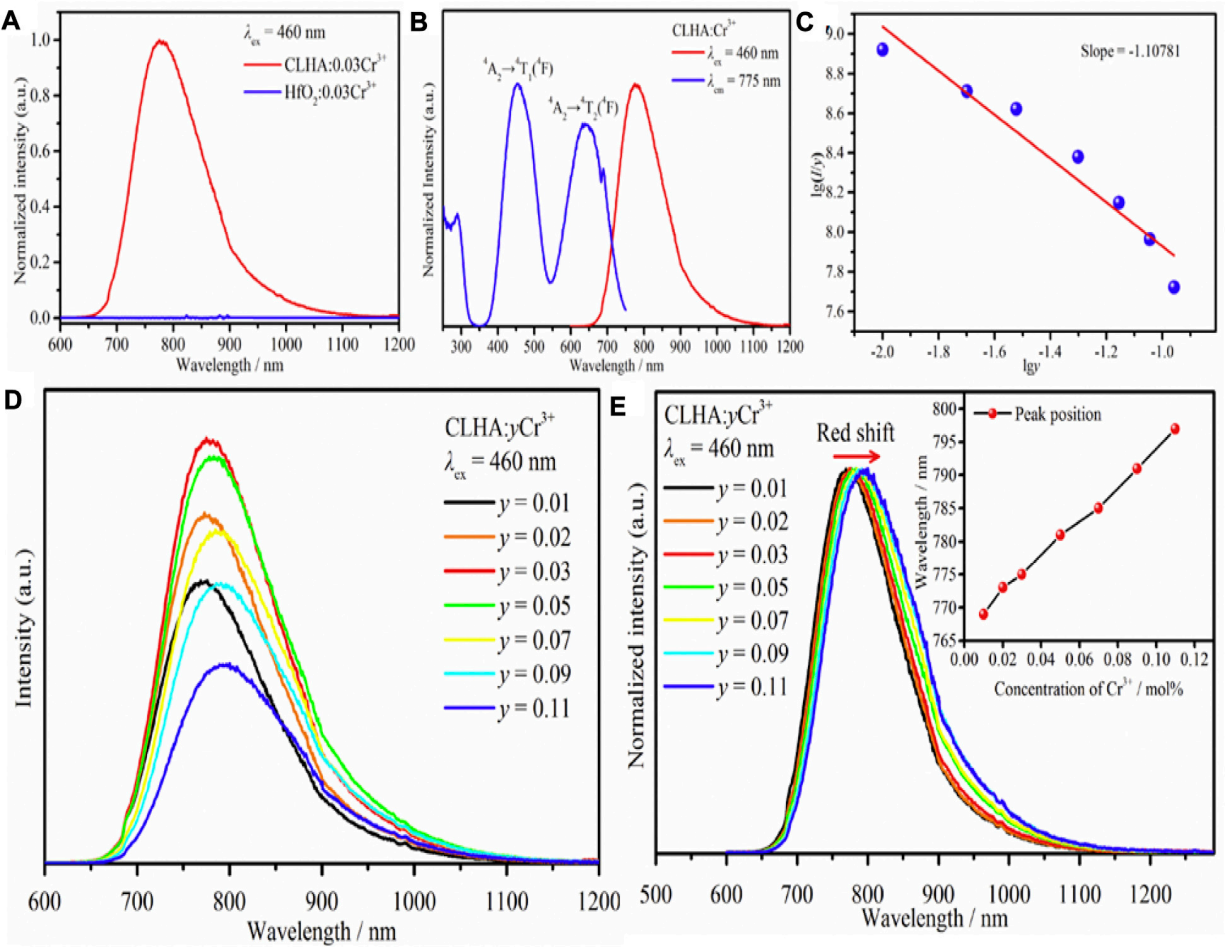
**(A)** CLHAO:0.03Cr^3+^ and HfO_2_:0.03Cr^3+^ PL spectrum. **(B)** The spectrum of CLHAO: Cr^3+^ in PL and PLE. **(C)** Relations between *I*_g_(*I*/*y*) and lgy (0.01 ≤ *y* ≤ 0.11). **(D)** The PL spectrum of CLHAO:*y*Cr^3+^ (0.01 ≤ *y* ≤ 0.11) phosphors. **(E)** Normalized the PL spectrum of CLHAO:*y*Cr^3+^ (0.01 ≤ *y* ≤ 0.11) under 460 nm excitation (Wu et al., 2021). Copyright 2021, Elsevier.

Figure 4A shows CLHAO:0.03Cr^3+^ and HfO_2_:0.03Cr^3+^ phosphors PL spectra. The instrument causes a small peak at 800–900 nm, while the emission intensity is zero in the impurity phase. In this case, the HfO_2_ impurity does not affect the photoluminescence features of CLHAO:Cr^3+^ phosphors. The PLE and PL spectra for CLHAO:0.03Cr^3+^ are shown in Figure 4B. The PL spectrum displays a wide emission band with an FWHM of 140 nm, covering the wavelength range of 650–1150 nm because of the ^4^T_2_→^4^A_2_ transition. A distinct peak with a center wavelength of 689 nm may also be seen. This is attributed to the transitions from ^4^A_2_ to ^3^E, called the R-line. The transitions between ^4^A_2_ and ^4^T_1_, ^4^A_2_ and ^4^T_2_, and ^4^A_2_ and ^4^T_2_ are each responsible for one of the three PLE bands with a central wavelength of 775 nm. Based on the PLE spectrum, it is evident that blue light can effectively excite the CLHAO:Cr^3+^ phosphor, which corresponds to the blue LED chips. Figure 4C shows that the emission intensity rises with rising Cr^3+^ concentrations, peaks at *y* = 0.03, and falls with rising *y*, indicating quenching will occur at this value of *y*. Cr^3+^ ions primarily generate this via nonradiative energy transfer, which includes exchange contact, radiation absorption, and multipolar interaction. To calculate the critical separation between Cr^3+^ ions in CLHAO phosphors, the line slopes of Iog(*I*/*y*) and log are 1.10781, as shown in Figure 4D. This value, which equates to around 6, implies that dipole-dipole interaction is required for quenching the CLHAO:Cr^3+^ concentration. Additionally, the PL spectra of CLHAO:*y*Cr^3+^ phosphors (*y* = 0.01, 0.02, 0.03, 0.05, 0.07, 0.09, and 0.11) triggered by 460 nm blue light excitation exhibit a shift from 769 nm for CLHAO:0.01Cr^3+^ to 797 nm for CLHAO:0.11Cr^3+^ is shown in Figure 4E.

### 3.3 Occupation of Tb^3+^ in multiple crystallographic sites

The trivalent Tb^3+^ ions are extensively used as dopant because of their strong line emission in the green spectral region (at a wavelength of roughly 543 nm) caused by the ^5^D_4_→^7^F_J_ transition (J = 3, 4, 5, and 6). However, they have drawbacks, such as the ultraviolet (UV) and near-UV absorption spectra of Tb^3+^ ions being very low because of the spin-forbidden character of their 4f→4f transitions. As a result of their poor luminous strength and absorption efficiency, this significantly restricts their usage in white LEDs. Therefore, green phosphors that have solely been doped with Tb^3+^ ions are typically inappropriate for use in white LEDs.

Energy transfer (ET) is a method that may be used to boost the brightness of Tb^3+^ ions by using some of the energy from the spin-allowed absorption of Eu^2+^ and Ce^3+^ ions. Despite the potential for improving the absorption spectrum by co-doping Eu^2+^ and Tb^3+^ ions, the Eu^2+^/Tb^3+^ pair has several clear restrictions that harm the luminescence quality and applications of Tb^3+^-triggered phosphors. (1) Eu^2+^ ions normally have an excitation band outside the visible range; (2) Tb^3+^ doping concentrations in co-doped phosphors with Eu^2+^/Tb^3+^ ions are usually low. Consequently, full Tb^3+^ green emissions are not possible. (3) When Eu^2+^ and Eu^3+^ ions are present together in phosphors co-doped with Eu^2+^/Tb^3+^ ions, Eu^3+^ ions can kill Eu^2+^ ion luminescence, reducing light output. The high absorption efficiency of Ce^3+^ ions in the near-ultraviolet range makes up for the absence of substantial absorption of Tb^3+^ ions in the area. This is because Ce^3+^ ions have a spin-allowed 4f →5d transition. The PL and PLE spectra of the phosphors made from CLHAO: 0.5Tb^3+^, further explained in the energy transfer (ET) section.

### 3.4 Energy transfer in CLHAO phosphors

Solid-state lighting, erasable optical data storage, and temperature sensors are just a few of the many uses that inorganic materials might be put to. This potential is demonstrated by the ability to achieve broadband, adjustable, and tunable emission via various energy transfer processes assisted by various co-dopant activators.

Tb^3+^ ions are often utilized as the light-emitting core of green-emitting phosphors due to their high green light emission (at around 543 nm), which is filled by electronic transitions from ^5^D_4_→^7^F_J_ (J = 3, 4, 5, and 6) (Xiao et al., 2017). On the other hand, these phosphors doped with Tb^3+^ ions have a distinct disadvantage. Because Tb^3+^ ions have spin-forbidden 4f → 4f transitions, their near-UV absorption spectra are very weak, and their poor absorption efficiency and luminous intensity limit their practical use in white LEDs (Chen and Wang, 2019; Vijayakumar et al., 2021). Hence, green phosphors containing just Tb^3+^ ions are typically incompatible with white LEDs (Yan et al., 2019).

**FIGURE 5:**
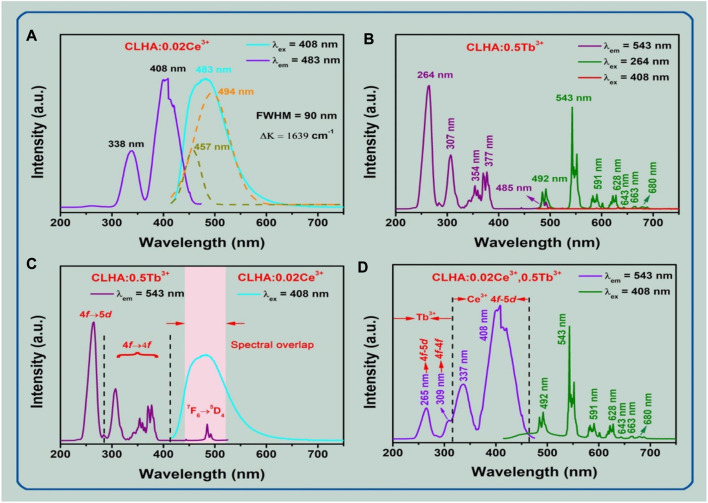
**(A)** PL and PLE spectra of CLHAO:0.02Ce^3+^ phosphors, with λ_ex_ = 408 nm and λ_em_ = 483 nm, respectively. **(B)** PL (λ_ex_ = 264 and 408 nm) and PLE (λ_em_ = 543 nm) spectra of CLHAO:0.5Tb^3+^ phosphors. **(C)** There is a considerable overlap between the PL spectrum of the CLHAO:0.02Ce^3+^ (λ_ex_ = 408 nm) and the PLE spectrum of the CLHAO:0.5Tb^3+^ (λ_em_ = 543 nm) phosphors. **(D)** The CLHAO phosphors’ PL and PLE spectra at 408 nm and 543 nm for 0.02Ce^3+^ and 0.5Tb^3+^, respectively, (Ma et al., 2021). Copyright 2021, Elsevier.

Various CLHAO:Ce^3+^/Tb^3+^ co-doped phosphors were developed with high temperature solid-state reactions. The PL and PLE spectra of the CLHAO: 0.02Ce^3+^ single doped phosphor is shown in Figure 5A (Ma et al., 2021). The CLHAO: 0.02Ce^3+^ phosphors exhibit a broad excitation band ranging from 300 to 470 nm due to the 4f→5d electronic transition of Ce^3+^ ions. The peak excitation occurs at 408 nm, with a secondary peak at 348 nm (Luo and Xia, 2014; Jiao et al., 2020). By using CLHAO: 0.02Ce^3+^ phosphors, a broad spectrum of high-intensity PL was produced. This PL has an emission peak at 483 nm due to the spin-allowed 5d→4f transition of Ce^3+^ ions (Sun et al., 2016; Yang et al., 2016).

The emission band, full width at half maximum (FWHM), was discovered to be 90 nm. The PL spectrum is surrounded by two bands that suit a Gaussian distribution. Ce^3+^ ion ^5^d→ ^2^F_5/2_ and ^5^d → ^2^F_7/2_ transitions produced 457 nm and 494 nm PL peak wavelengths (21881 cm^-1^ and 20242 cm^-1^, respectively) (Zhou et al., 2017). The calculated energy level difference between ^2^F_5/2_ and ^2^F_7/2_ is 1639 cm^-1^, which is extremely close to the expected value of 2000 cm^-1^ (Setlur et al., 2006). The PLE and PL spectra of the CLHAO:0.5Tb^3+^ phosphors are displayed in Figure 5B. When measured at 543 nm, the largest excitation band was identified at 264 nm in the 240–280 nm range. This happened because of the Tb^3+^ ions’ spin-permitted 4f →5d transition (Huang et al., 2017; Guo et al., 2018; Li et al., 2018; Jia et al., 2020). With peak wavelengths of 307, 354, 377, and 485 nm, the PLE spectrum also exhibits weak excitation peaks between 290 and 500 nm. These peak wavelengths were assigned to the Tb^3+^ ions’ individual ^7^F_6_→ ^5^H_7_, ^7^F_6_→ (^5^L_9_, ^5^G_4_), ^7^F_6_→ ^5^G_6_, and ^7^F_6_ →^5^D_4_ transitions (Vijayakumar et al., 2019; Li et al., 2020).

The near-UV PLE spectrum was produced by Tb^3+^ ions when they were doped singly. Still, because their 4f→4f transitions were spin-prohibited and their absorption intensity was incredibly low, their usage in near-UV-excited white LEDs was severely constrained. The CLHAO:0.5Tb^3+^ garnet phosphors emit green light when illuminated at 264 nm, and the PL spectra show a series of strong emission peaks at 492, 543, 591, 628, 643, 663, and 680 nm, which correspond to the ^5^D_4_→ ^7^F_6_, ^5^D_4_→ ^7^F_5_, 5D_4_ →^7^F_3_, 5D_4_ →^7^F_2_, 5D_4_→ ^7^F_1_, and ^5^D_4_→F_0_ (Hua et al., 2019; Zhang et al., 2020). As can be observed, the PL spectrum was dominated by the green emission at 543 nm. The PL spectrum of the optimized CLHAO:0.5Tb^3+^ phosphors was measured under 408 nm excitation. The results were plotted in Figure 5B, which confirms the line emission in the green spectral region.

The PLE and PL of CLHAO with single-doped Ce^3+^ and Tb^3+^ were measured to shed light on the energy transfer mechanism. The results were plotted in Figure 5C, illustrating that strong overlapping was observed in the 475–520 nm wavelength region. The standard CLHAO:0.02Ce^3+^,0.5Tb^3+^ phosphors’ PLE (*λ*_em_ = 543 nm) and PL (*λ*_ex_ = 408 nm) spectra are shown in Figure 5D. The spin-allowed 4f → 5d transitions of the Ce^3+^ ions create a robust and broad excitation band with a peak at 408 nm and a shoulder at 337 nm, as seen in the PLE spectra. The 4f → 5d and ^7^F_6_ → ^5^H_7_ transitions of Tb^3+^ ions were also assigned to cause the very low excitation peaks at 265 and 309 nm.

The distinctive excitation band Ce^3+^ ions lead to the highly efficient green emission of CLHAO:0.02Ce^3+^,0.5Tb^3+^ co-activated phosphors, confirming the efficient energy transfer from the trivalent Ce^3+^ to Tb^3+^ ions in the CLHAO phosphors host lattice. The CLHAO:0.02Ce^3+^,0.5Tb^3+^ phosphors generated a dazzling green light with a definite emission peak at 543 nm when stimulated at 408 nm. Figure 5D displays the Tb^3+^ ion transitions as a series of strong emission peaks at 492, 543, 591, 628, 643, 663, and 680 nm. There are also ^5^D_4_ →^7^F_6_, ^5^D_4_ →^7^F_5_, ^5^D_4_ →^7^F_4_, ^5^D_4_→ ^7^F_3_, ^5^D_4_ →^7^F_2_, ^5^D_4_ →^7^F_1_, and ^5^D_4_ →^7^F_0_ in addition to ^5^D_4_→ ^7^F_6_. Based on these findings, co-doping with Ce^3+^ ions may allow Tb^3+^ ions to expand their near-UV absorption band. As a result, when exposed to near-UV light, the CLHAO:0.02Ce^3+^,0.5Tb^3+^ phosphors work well as green components to produce white LEDs.

**FIGURE 6:**
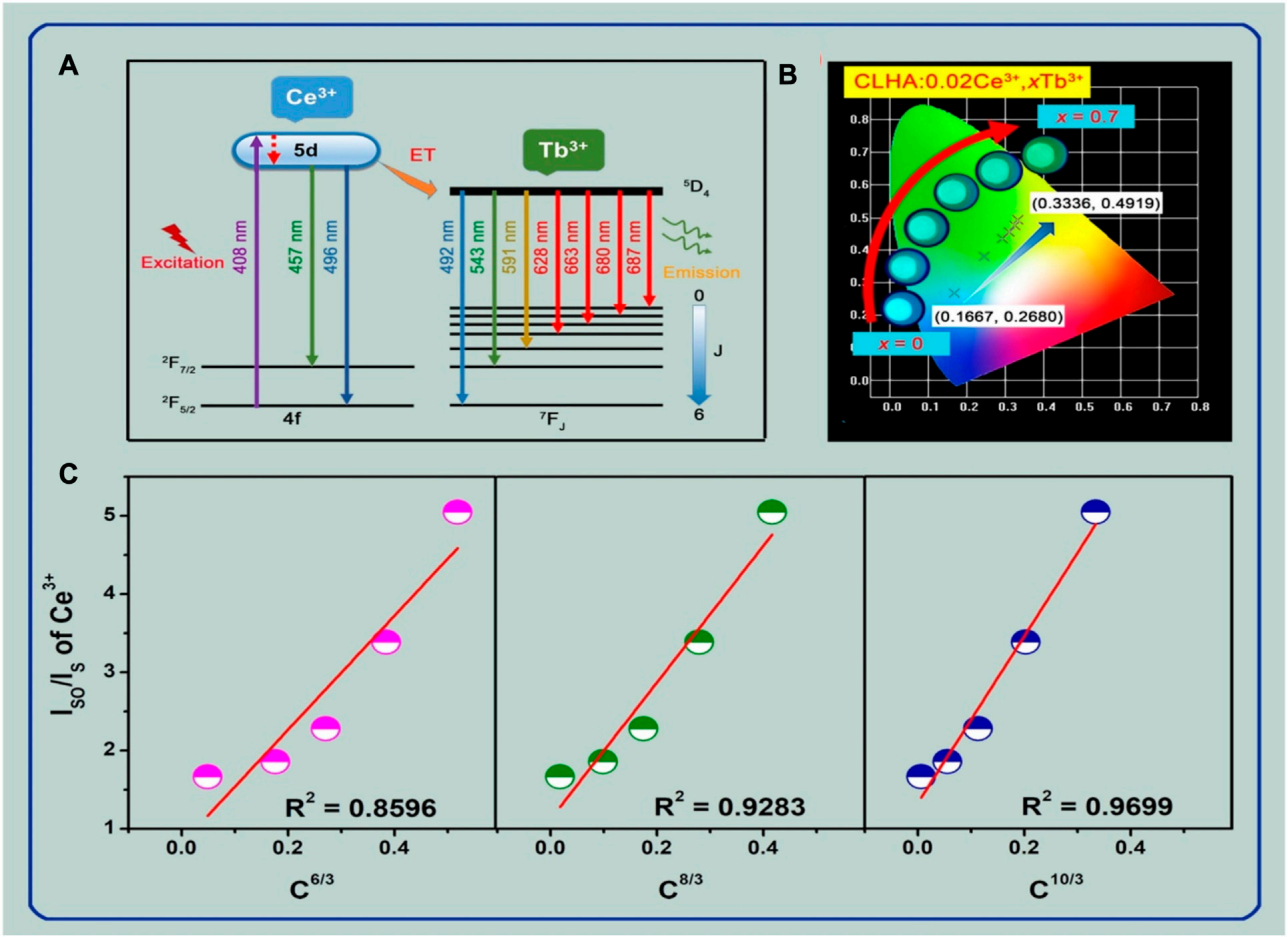
The color-tunable emission of the CLHAO phosphors activated at 408 nm, which spans from cyan to green, is shown in **(A)**, along with an example of the Ce^3+^, Tb^3+^ energy transfer ET mechanism. **(B)** The CLHAO:0.02Ce^3+^, *x*Tb^3+^, (*x* = 0, 0.2, 0.4, 0.5, 0.6, and 0.7) CIE chromaticity diagram of these phosphors, as well as digital photographs of them taken with a 365 nm UV light. **(C)** The CLHAO:0.02Ce^3+^, *x*Tb^3+^ phosphors (*x* = 0, 0.2, 0.4, 0.5, 0.6, and 0.7) enhanced by 408 nm light, as well as digital photos of these phosphors taken with a 365 nm UV lamp (Ma et al., 2021). Copyright 2021, Elsevier.

Figure 6A illustrates the energy transfer (ET) mechanism from the trivalent Ce^3+^ to the co-doped Tb^3+^ ions in CLHAO:Ce^3+^, Tb^3+^ co-activated phosphors. The electron shifts to a 5d excited state from ^2^F_5/2_ of Ce^3+^ ions absorbing photons at 408 nm wavelengths. Excited electrons from the most excited 5d state moved via non-radiative transitions to reach the lowest excited 5d state. A blue emission with a peak at 483 nm was produced when the electrons of the lowest 5d excited state (Ce^3+^ ions energy levels) changed back to their 4f ground state. Using the ET method, the lower 5d excited state electrons of other Ce^3+^ ions transferred their energy to the Tb^3+^ ions’ ^5^D_4_ excited state in the interim. The excited ^5^D_4_ state electrons in the Tb^3+^ ions were released after being radiated back to the ground state ^7^F_J_ (J = 1→6), which led to a series of recognizable vivid green emissions because of the ^5^D_4_→^7^F_J_ (J = 1→6) transitions. Figure 6B shows the CIE chromaticity diagram for the appropriate phosphors. The increasing of the Tb^3+^ doping concentrations makes it possible to change the CIE color coordinates of CLHAO:0.02Ce^3+^, *x*Tb^3+^ phosphors from cyan (0.1667, 0.268) to green (0.3336, 0.4919) due to the efficient Ce^3+^, Tb^3+^ ET process. Aside from that, the CLHAO:0.02Ce^3+^, *x*Tb^3+^ digital pictures of (*x* = 0, 0.2, 0.4, 0.5, 0.6, and 0.7) phosphors driven by 365 nm light are given in Figure 6B to illustrate the color-tunable emission of cyan to green. The CLHAO:0.02Ce^3+^ phosphors and the CLHAO:0.02Ce^3+^, *x*Tb^3+^ (*x* = 0.2, 0.4, 0.5, 0.6, and 0.7) phosphors’ intensity values of Ce^3+^ ions at 457 nm are expressed by *I*_S0_ and *I*_S_, respectively. C indicates the overall concentration of Ce^3+^ and Tb^3+^ ions, and the electric multipolar contact type indicated by *n* during energy transfer ET, where *n* = 6, 8, and 10 are, respectively, connected to dipole-dipole, dipole-quadruple, and quadruple-quadruple interactions. Finally, as shown in Figure 6C, the predicted values of *I*_S0_/*I*_S_ are reliant on the line fitting results of $C^{\frac{6}{3}}$ , $C^{\frac{8}{3}}$ , and $C^{\frac{10}{3}}$ . The fitting parameters *R* ^2^ for n=6,8 and 10 were 0.8596, 0.9283, and 0.9699, respectively. This indicates that the Ce^3+^→Tb^3+^ energy transfer ET process in the CLHAO:Ce^3+^→Tb^3+^ phosphors were a quadruple-quadruple interaction mechanism as the best linear fitting relationship between *I*_S0_/*I*_S_ and C^n/3^ was found at *n* = 10.

**FIGURE 7:**
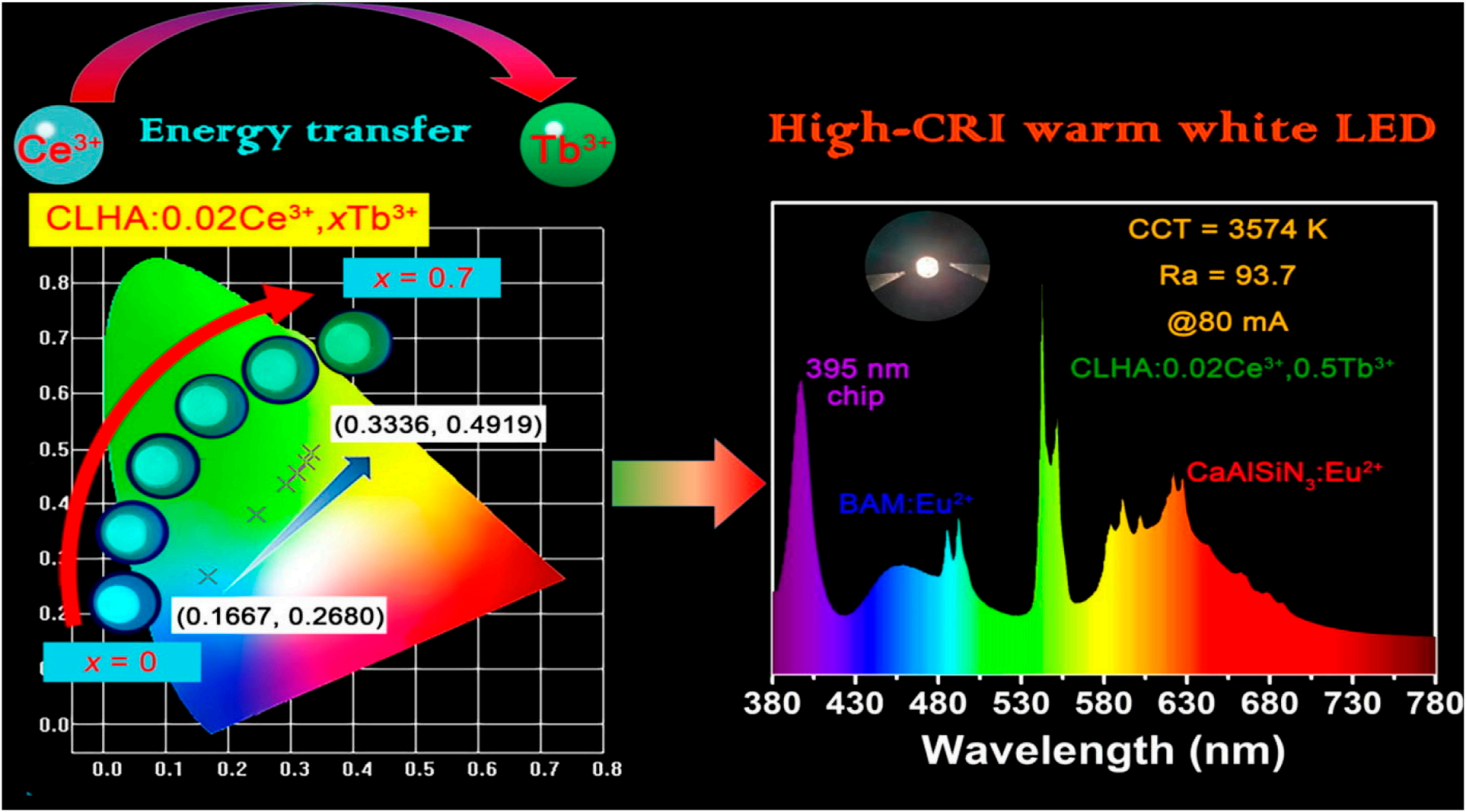
The 0.02Ce^3+^, *x*Tb^3+^ CIE chromaticity diagram is shown in the following examples: CLHAO: 0.02Ce^3+^, *x*Tb^3+^(*x* = 0, 0.2, 0.4, 0.5, 0.6, and 0.7) phosphors lighted by 408 nm light, as well as digital images of the phosphors obtained with a warm white LED with a high CRI and a 365 nm UV lamp (Ma et al., 2021). Copyright 2021, Elsevier.

We also determined the CIE color coordinates for the CLHAO:0.02Ce^3+^, *x*Tb^3+^ phosphors with (*x* = 0, 0.2, 0.4, 0.5, 0.6, and 0.7) based on their PL spectra at 408 nm. Figure 7 illustrates the matching CIE diagram of these phosphors. We discovered that the CLHAO phosphors’ CIE color coordinates changed from cyan (0.1667, 0.2680) to green (0.3336, 0.4919) when the quantities of Tb^3+^ doping rose. This change may be attributed to the effective energy transfer between Ce^3+^→Tb^3+^. The CLHAO:Ce^3+^,Tb^3+^ garnet phosphors exhibit high Ce^3+^→Tb^3+^ energy transfer and generate green near-UV light. A solid-state procedure carried out at a high temperature was used to create the green phosphor CLHAO:Ce^3+^,Tb^3+^. Between Ce^3+^ and Tb^3+^ ions, quadruple-quadruple interactions have been linked to the ET process. The best green phosphors have internal and exterior quantum efficiencies of 77.1% and 55.8%, respectively, for CLHAO:0.02Ce^3+^,0.5Tb^3+^ phosphors compositions.

To construct a white LED device with near-UV pumping, CLHAO:0.02Ce^3+^, 0.5Tb^3+^ phosphors were employed as green-emitting color converters. Figure 7 demonstrates that the LED device produced a dazzling warm-white light with a high color rendering index (93.7), a low associated color temperature (3574 K) with CIE chromaticity coordinates (0.3922, 0.3633), and a greater luminous efficacy (29.35 lm/W) at 80 mA.

### 3.5 Applications of white LEDs

To determine the possibility of CLHAO:Ce^3+^ phosphors to fulfil the cyan color gap in the fabrication of white LEDs for the applications of solid-state lighting, a white LED device was fabricated with the addition of the prepared cyan emission with the combination of blue, green, and red phosphors. The broad emission band of CLHAO:Ce^3+^ cyan emitting phosphors effectively filled the cyan gap, which led to a white light generation with high CRI values (Ra = 89.4, R9 = 49.5, and R12 = 81.8) that were noticeably higher than those of red, green, and blue phosphors converted LED (Ra = 83.2, R9 = 11.5, and R12 = 70.7).

**FIGURE 8:**
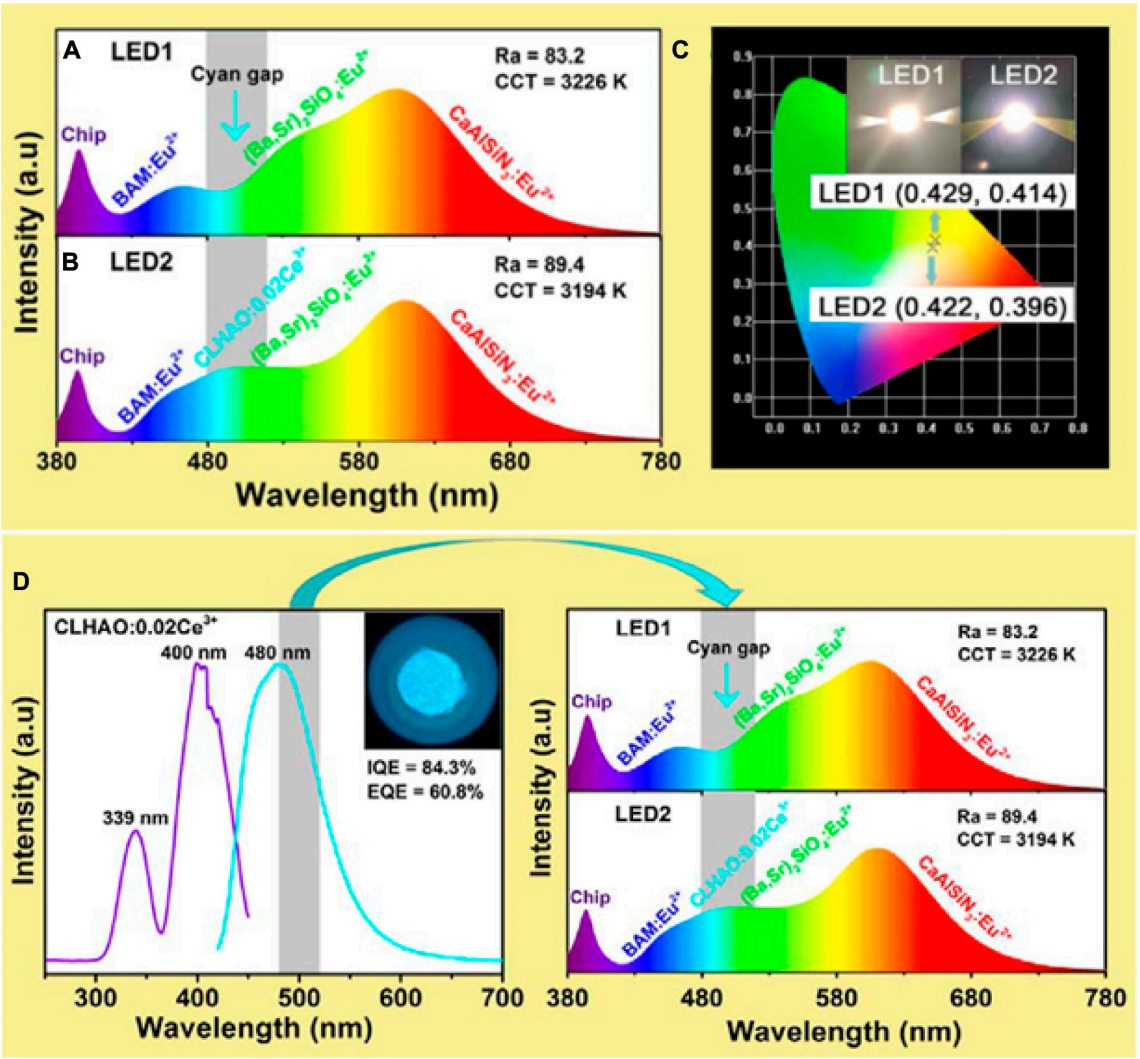
The spectra of **(A)** LED1 device and **(B)** LED2 device that are both driven by 120 mA. **(C)** Digital images of 120 mA-powered LED1 and LED2 devices with CIE chromaticity coordinates. **(D)** Cyan emitting phosphors CLHAO:0.02Ce^3+^ (Zhang Z. J. et al., 2021). Copyright 2021, Elsevier.

These two white LEDs produced bright, warm white light when each was supplied with 120 mA. Figures 8A, B displays the emission spectra of these devices. The LED1 (RGB phosphors converted) device’s emission spectrum seemed to have a cyan gap between 480 and 520 nm Figure 8A. The broad emission band of CLHAO:Ce^3+^ phosphors effectively filled the cyan gap, and the CRI values of LED2 (blue, cyan, green, and red phosphors-based device) (Ra = 89.4, R9 = 49.5, and R12 = 81.8) were noticeably higher than those of LED1 (Ra = 83.2, R9 = 11.5, and R12 = 70.7). Compared to LED1, LED2’s CCT value (3,194 K) was very similar (3,226 K).

Digital images and CIE chromaticity graph coordinates for the LED1 and LED2 devices are shown in Figure 8C. LED1 and LED2 have estimated CIE colored coordinates of (0.429, 0.414) and (0.422, 0.396), respectively. LED2 illumination was closer to white light. The evidence is overwhelming that CLHAO: 0.02Ce^3+^ cyan phosphors hold enormous promise for usage in high-color-rendering white LEDs. Figure 8D illustrates that the Ca_2_LuHf_2_Al_3_O_12_:0.02Ce^3+^ cyan phosphors that are near UV excitable and have internal quantum efficiency (IQE) and external quantum efficiency (EQE) values of 84.3% and 60.8%, respectively, are efficient for filling the cyan gap and producing white LEDs with outstanding color rendering.

## 4 Tuning of PL with different cations substitution

Aside from developing broadband cyan emission to fulfill the cyan gap in RGB phosphors converted white-LEDs, the emission spectrum of CLHAO:Ce^3+^ garnet phosphors can be efficiently tuned in the cyan and green color for the desired spectral region. More specifically, phosphor materials with extraordinary photoluminescence capabilities must be created for the subsequent development of high-quality solid-state white illumination. The broadband cyan-emitting phosphor is crucial to achieve “full-visible-spectrum lighting” and close the spectral gap since the emission spectrum of conventional phosphor-converted (w-LEDs) comprises a blue-green cavity. The synthesis of the thermally stable cyan-emitting Ca_2_YHf_2_Al_3_O_12_:Ce^3+^ garnet phosphor was doped with Ce^3+^. The prepared CYHAO: xCe^3+^ phosphor excitation band spans a wide range of wavelengths, from 360 to 460 nm, with a maximum peak at 408 nm. As a result, it can work with an LED chip that produces near-ultraviolet (NUV), which has a wavelength shorter than 400 nm and is produced by an LED chip. The best sample of CYHAO: 0.03Ce^3+^ showed robust broadband cyan emission when irradiated at 408 nm. The wavelength and bandwidth of the emission were 493 nm and 100 nm, respectively. The sample has a high internal quantum efficiency (IQE) of 89.5% despite having a low external quantum efficiency (EQE) of just 69.1% (Jia et al., 2020).

**FIGURE 9:**
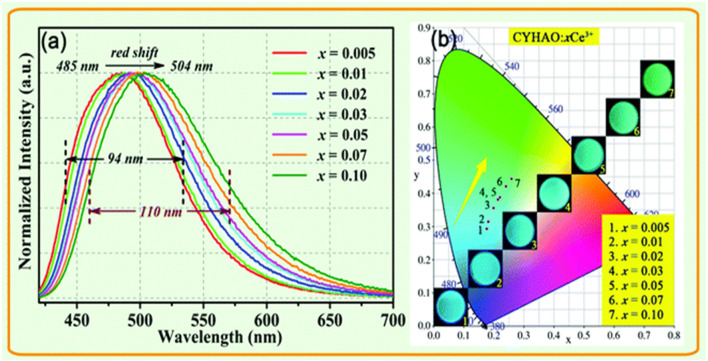
**(A)** Normalized photoluminescence (PL) spectrum of CYHAO:*x*Ce^3+^ phosphors excited at 408 nm. **(B)** The CIE diagram demonstrating the chromaticity of CYHAO:*x*Ce^3+^ samples. The associated pictures of samples obtained with a UV light emitting 365 nm (Jia et al., 2020). Copyright 2020, Royal Society of Chemistry.

The normalized photoluminescence emission spectrum of CYHAO:*x*Ce^3+^ garnet phosphors stimulated at 408 nm was plotted in Figure 9A to show the influence of Ce^3+^ ions in the PL characteristics. Naturally, the emission peak wavelength and the bandwidth (FWHM) varied as the concentration of Ce^3+^ doping increased. The dominant peak of the photoluminescence (PL) was observed at 485 nm at x = 0.005 and moved to 504 nm at x = 0.10, moving the peak point 19 nm toward the longer wavelength. The following is an explanation for the phenomenon of redshift.

The smallest Y^3+^ ions (*r* = 1.019 Å) were replaced by the bigger Ce^3+^ ions (*r* = 1.143 Å) in the phosphors CYHAO:*x*Ce^3+^. The Ce^3+^-O^2-^ bond may be compressed in the hard garnet structure when the Ce^3+^ concentration increases due to a possible reduction in the distance between ligands and light centers. An increase in Ce^3+^ 5d crystal field splitting thus caused the red shift in emission spectra. There was also the possibility that the Ce^3+^ activators might transfer energy, which would explain the redshift. An increase in low-energy emission and a shift of the emission maxima to a longer wavelength were ultimately caused by a greater energy transfer from higher 5d level Ce^3+^ ions to lower-level Ce^3+^ ions as the Ce^3+^ concentration enhanced. The emission bands of CYHAO:*x*Ce^3+^ phosphors also grew wider when the Ce^3+^ doping concentration enhanced from *x* = 0.005 to *x* = 0.10. Figure 9B displays the CIE chromaticity diagram of the CYHAO:*x*Ce^3+^ garnet phosphors. By increasing the concentration of Ce^3+^ ions from x = 0.005 to x = 0.10, it is possible to modify the emission colors from cyan to green with CIE chromaticity coordinates ranging from (0.1756, 0.2936) to (0.1756, 0.2936), (0.2591, 0.4438), and so on. The inset of Figure 9B depicts digital photographs of these phosphors acquired under 365 nm UV light, showcasing their robust emission and range of emission colors.

Similarly, a potential CaLa_1-*x*_Lu_*x*_HAO:Ce^3+^ garnet phosphor has been created based on the solid-solution design of chemical cation substitution in Ca_2_La_1-*x*_Lu_*x*_Hf_2_Al_3_O_12_:Ce^3+^ garnet phosphors (Chan et al., 2023). The strategy of cationic substitution discussed here can create a new path towards developing high-efficiency luminescent materials by modifying the crystal structure. This approach will also significantly and broadly impact solid-state white lighting (Li et al., 2008; Tolhurst et al., 2017; Yan et al., 2017; Leaño et al., 2018; Wang et al., 2019; Amachraa et al., 2020; Ding et al., 2021; Viswanath et al., 2021; Chan et al., 2022a; Chan et al., 2022b; Wang et al., 2022; Yang et al., 2022; Li et al., 2023).

**FIGURE 10:**
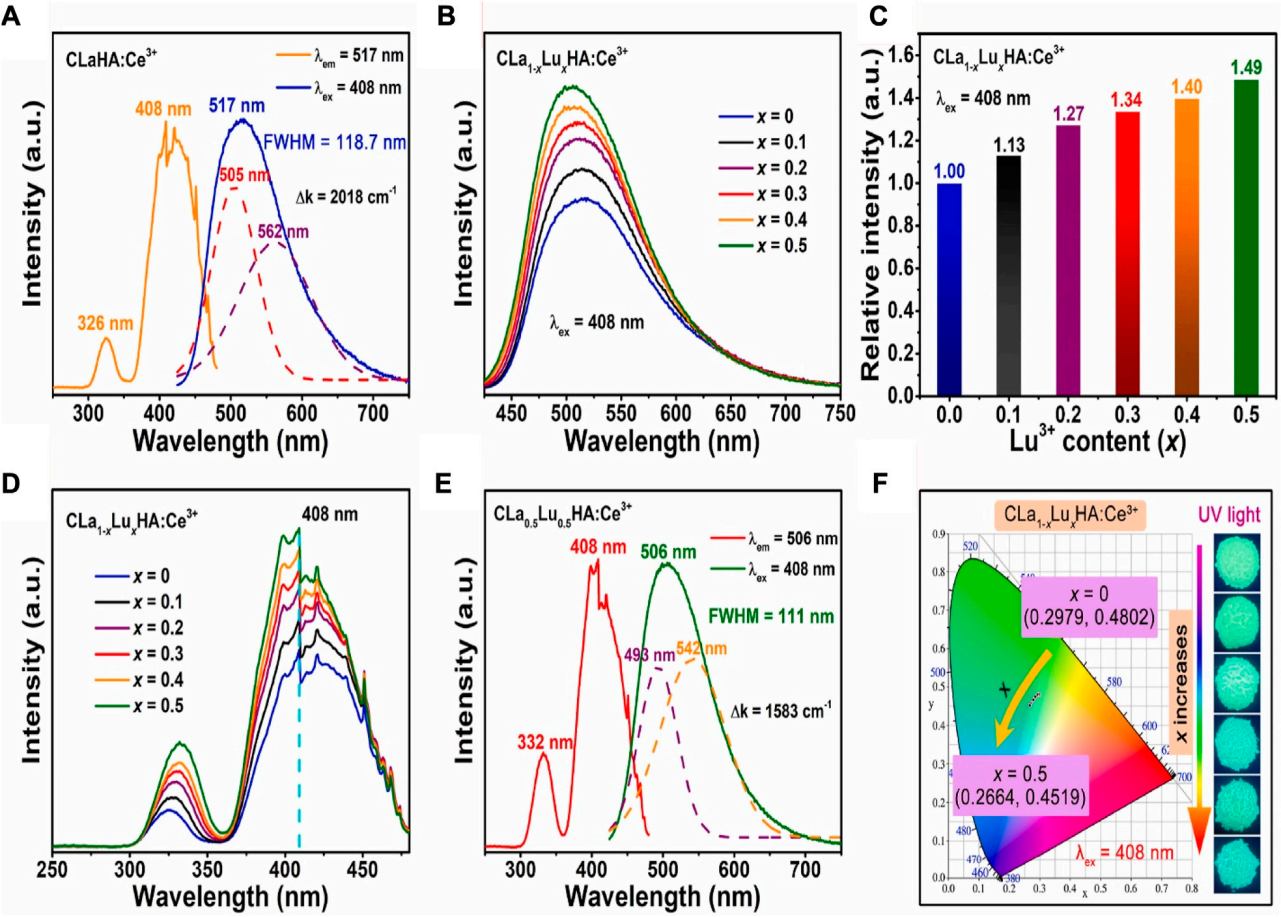
**(A)** PLE and PL spectra for the sample CLaHAO:Ce^3+^. **(B)** PL spectra for 408 nm-excited CLa_1-*x*_Lu_*x*_HAO:Ce^3+^ phosphors. **(C)** CLa_1-*x*_Lu_*x*_HAO:Ce^3+^ phosphors’ relative integrated PL intensity. **(D)** PLE spectra for CLa_1-*x*_Lu_*x*_HAO:Ce^3+^ phosphors. **(E)** PLE and PL spectra for the optimized CLa_0.5_Lu_0.5_HAO:Ce^3+^ phosphors. **(F)** CIE chromaticity diagrams for CLa_1-*x*_Lu_*x*_HAO:Ce^3+^ phosphors (λ_ex_ = 408 nm); insets show images taken with a 365 nm UV light of the CLa_1-*x*_Lu_*x*_HAO:Ce^3+^ phosphors (Chan et al., 2023). Copyright 2023, Elsevier.

The trivalent Lu^3+^ ions may significantly improve luminous performance in CLa_1-*x*_Lu_*x*_HAO:Ce^3+^ phosphors when La^3+^ ions are substituted for Lu^3+^ ions. Thus, CLa_1-*x*_Lu_*x*_HAO:Ce^3+^ phosphors have been thoroughly investigated for their luminous characteristics. The PLE and PL spectra of the CLaHAO:Ce^3+^ phosphor sample are shown in Figure 10A without Lu^3+^ doping. In the region of 300–350 nm wavelengths, with a peak at 326 nm, the PLE spectrum obtained at 517 nm contains a weak excitation band. In the region 350–480 nm spectral range, there is an intense broad excitation band with a peak at 408 nm. This transition might be attributed to Ce^3+^ ions’ transition from the ground state 4f to their excited ^5^d_2_ and ^5^d_1_ states, which permit spin and parity (Hakeem et al., 2018; Li et al., 2022).

With an excitation wavelength of 408 nm, the CaLaHAO:Ce^3+^ phosphor emits a strong, broad-band green emission up to 517 nm with a full width at half maximum (FWHM) of 118.7 nm. Two Gaussian sub-bands in the CaLaHAO:Ce^3+^ phosphor’s PL emission band correspond to ion transitions caused by Ce^3+^ ions ^5^d→^2^F_5/2_ and ^5^d→^2^F_7/2_, respectively, (Leaño et al., 2018; Chan et al., 2022a). The energy difference (∆k) between the two Gaussian bands has been calculated to be 2018 cm^−1^, which is close to the theoretical difference of 2000 cm^−1^ and indicates that the Ce^3+^ ions only have one site in the host lattice of Ca_2_LaHf_2_Al_3_O_12_ (Yan et al., 2017; Chan et al., 2022b).

The PL spectra of CaLa_1-*x*_Lu_*x*_HAO: Ce^3+^ (0 ≤ *x* ≤ 0.5) phosphors upon 408 nm excitation are shown in Figure 10B. In all these samples, cyan-green emission bands are brilliant and broad in the range of 425–750 nm, with a slight blue shift occurring at the emission peak location when Lu^3+^ concentration increases (517 nm at *x* = 0–506 nm at *x* = 0.5). Figure 10C shows Lu^3+^ concentration-dependent integrated PL intensity of CaLa_1-*x*_Lu_*x*_HAO:Ce^3+^ phosphors. Figure 10D shows the PLE spectra of CaLa_1-*x*_Lu_*x*_HAO:Ce^3+^ (0 ≤ *x* ≤ 0.5) phosphors. Each of them consists of two bands of excitation. With a peak at 408 nm (caused by the Ce^3+^ ion’s 4f–5d^1^ transition), these samples exhibit a broad and strong excitation band in the 350–480 nm spectral range. The intensity of excitation increases as the Lu^3+^ content (*x*) increases. This indicates that near-UV LED chips can function effectively in the excitation of CaLa_1-*x*_Lu_*x*_HAO:Ce^3+^ phosphors.

Moreover, all these CaLa_1-*x*_Lu_*x*_HAO:Ce^3+^ phosphors samples exhibit a relatively weak excitation band at 300–350 nm wavelengths (due to the 4f–5d^2^ transition of Ce^3+^ ions), and the intensity of this band increases with increasing Lu^3+^ content (x), but the excitation peak position red-shifts from 326 nm for *x* = 0–332 nm for *x* = 0.5. The CaLa_0.5_Lu_0.5_HAO:Ce^3+^ solid solution sample has the maximum emission intensity among the CaLa_1-*x*_Lu_*x*_HAO: Ce^3+^ (0 ≤ *x* ≤ 0.5) garnet phosphors. Figure 10E shows the PLE and PL spectra of the optimized CaLa_0.5_Lu_0.5_HAO:Ce^3+^ phosphors. As the PLE spectrum shows, Ce^3+^ ions exhibit spin-and-parity-allowed electronic transitions of 4f–5d^1^ and 4f–5d^2^ in the 300–480 nm regions. A prominent cyan-green emission band was observed upon stimulation at 408 nm in the CaLa_0.5_Lu_0.5_HAO:Ce^3+^ phosphors sample. In addition, the emission band can be split into two Gaussian-fitting bands at 493 nm and 542 nm, corresponding to the electronic transitions of Ce^3+^ ions at 5d→2F_5/2_ and 5d→2F_7/2_. According to the experimental results, the energy difference between 2F_5/2_ and 2F_7/2_ levels is close to the theoretically calculated value of 1583 cm^-1^ in CaLa_0.5_Lu_0.5_HAO:Ce^3+^ phosphors.

Figure 10F presents the CaLa_1-*x*_Lu_*x*_HAO:Ce^3+^ phosphors and their CIE chromaticity diagram. CIE chromaticity coordinates show a blue shift as Lu^3+^ concentration increases, going from (0.2979, 0.4802) for *x* = 0 to (0.2664, 0.4519) for *x* = 0.5. As *x* increases, the emission color of CaLa_1-*x*_Lu_*x*_HAO:Ce^3+^ becomes cyan, green, with the cyan component deepening. The PLE and PL characteristics of Ce^3+^-activated phosphor materials could be fine-tuned by adjusting their coordination environment.

**FIGURE 11:**
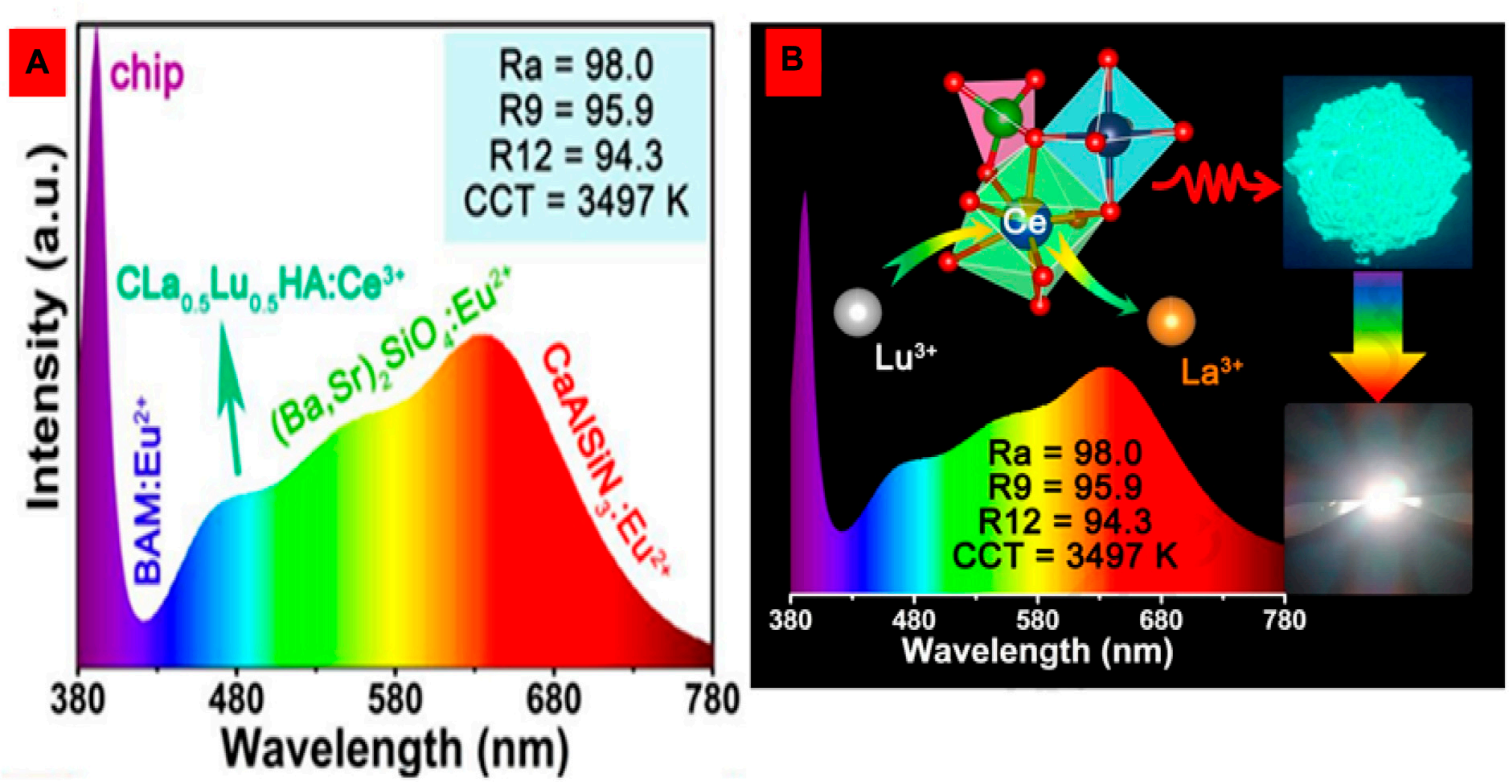
**(A, B)** The solid-solution phosphors Ca_2_La_1-*x*_Lu_*x*_Hf_2_Al_3_O_12_:Ce^3+^ with cyan and green emission showed highly symmetric crystal structures due to cation substitution (Chan et al., 2023). Copyright 2023, Elsevier.

For near-UV-pumped full-visible spectrum white LEDs with ultra-high color rendering indices (Ra = 98, R_9_ = 95.9, and R_12_ = 94.3), novel cyan-green phosphors with a superior quantum efficiency (76.4%) and significantly higher thermal stability have been developed. Due to the induced highly symmetric crystal structure, solid-solution phosphors synthesized from Ca_2_La_1-*x*_Lu_*x*_Hf_2_Al_3_O_12_:Ce^3+^ exhibit enhanced cyan-green emission with enhanced thermal stability for full-spectrum white LEDs, as shown in Figures 11A, B.

**FIGURE 12:**
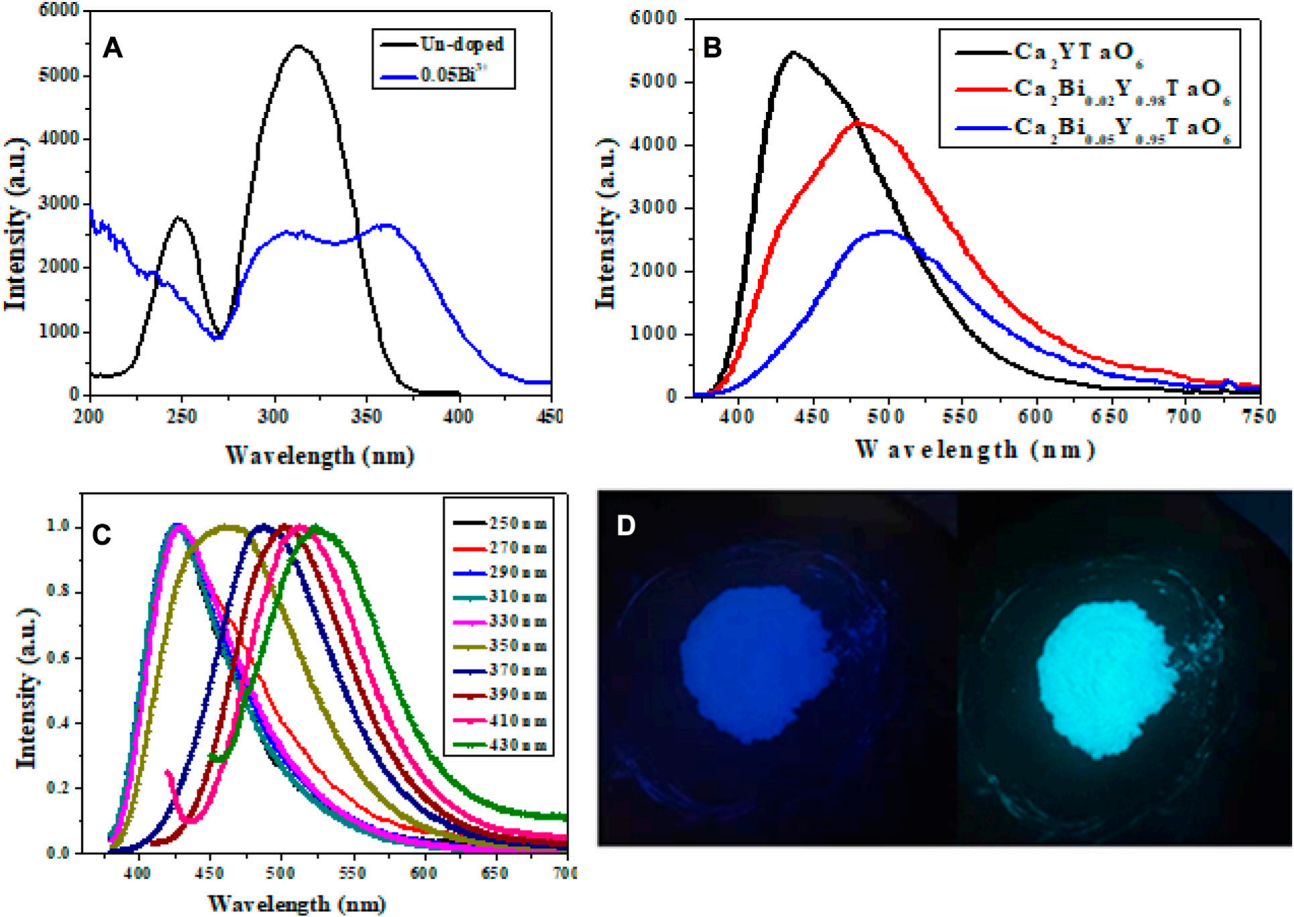
Ca_2_YTaO_6_ phosphor optical characteristics **(A)** PLE of un-doped and 0.05Bi^3+^, **(B)** PL of Bi^3+^ concentration samples, **(C)** PL of 0.02Bi^3+^ at various excitation wavelengths, and **(D)** the optimized Ca_2_YTaO_6_:0.02Bi^3+^ double perovskite phosphor sample as depicted in a digital image at 254 nm and 365 nm (Khan et al., 2021). Copyright 2021, Elsevier.

In our most recent study, Ca_2_YTaO_6_ confirmed that the different colors of light obtained from Ca_2_YTaO_6_:Bi^3+^ double perovskite oxide phosphors are caused by several luminescence centers. The smooth change in the emission spectrum from blue to cyan and green indicates several light sources. To investigate how the amount of Bi^3+^ concentration affects the emission of Ca_2_YTaO_6_:Bi^3+^ phosphors, we examined the PLE and PL spectra of the as-prepared samples at several monitored emission and excitation wavelengths at room temperature. Figure 12A compares the PLE spectra of the Bi^3+^ doped (monitored at 480 nm) and un-doped (424 nm) samples. It is clear from the comparison that the un-doped sample displayed a broadband excitation at the monitored wavelength of 424 nm, ranging from 200 nm to 400 nm, with the dominant peak around 315 nm on the high energy side.

In contrast, the PLE spectra of the Bi^3+^-doped Ca_2_YTaO_6_ phosphors show impressive broadness. In particular, the PLE spectra of the Bi^3+^-doped material expanded to longer wavelengths (at least 50 nm red-shifted). They gained an additional peak at 361 nm on the lower energy side because of the absorption of ^1^S_0_ to ^3^P_1_ in the activated Bi^3+^ ions. We investigate whether the presence of several luminescence centers in the host lattice of Ca_2_YTaO_6_ phosphors may explain the occurrence of multiple excitation peaks, in addition to varied excitation, samples with and without Bi^3+^ doping yield phosphors with distinct emissions (Khan et al., 2021).

The increase of Bi^3+^ concentration causes a noticeable broadening of the dominant emission peak from 424 nm to 480 nm–500 nm as illustrated in Figure 12B. To understand the reason behind the varied color emission of Ca_2_YTaO_6_:Bi^3+^ phosphors, we looked at the room-temperature photoluminescence (PL) of optimized Ca_2_YTaO_6_:0.02Bi^3+^ phosphors with various excitations (250–430 nm with a 20 nm spacing). The optimized Ca_2_YTaO_6_:0.02Bi^3+^ phosphors sample’s normalized PL spectra can be shown in Figure 12C, and they demonstrate that the emission spectrum has successfully been changed from blue to cyan and green, demonstrating the existence of many luminescence centers. The prepared Ca_2_YTaO_6_:0.02Bi^3+^ double perovskite oxide phosphors’ digital images acquired at 254 and 365 nm showed that the color tuning was effective at the two excitation wavelengths (Figure 12D).

## 5 Concluding remarks

In conclusion, phosphors with the general chemical formula Ca_2_LuHf_2_Al_3_O_12_ (CLHAO) are a significant component of the inorganic material family, where numerous cation substitutions may be performed to produce optimal compositions for application in various sectors of illumination. The concepts and techniques of (a) fulfilling of cyan gap in the full spectral region of white-LEDs, (b) cations substitution to accomplish efficient tuning of the emission color and (c) the growing and tuning abilities of sensitizer emission because of the efficient energy-transfer phenomena via doping using different rare-earth (RE) and transition metal (TM) ions, including Ce^3+^, Cr^3+^, Tb^3+^, and Bi^3+^, were thoroughly examined in this review. The choice of host materials is essential in producing w-LED phosphors and the doped activators. The interaction between the host materials and the doped activators alters the luminous features of this garnet phosphor.

A major challenge in garnet ceramics for solid-state lighting (SSL) is distinguishing between concentration quenching, thermal quenching, and optical excitation quenching (Yan et al., 2017; Khan et al., 2022; Khan et al., 2023a; Ali et al., 2023; Khan et al., 2023b). Investigating the multiple coupling effects among these mechanisms will drive future research. However, addressing the quenching effect of red-emitting ceramics with a longer decay time under high-power density laser excitation remains difficult. Selecting an excitation source is critical to pursuing high-quality and healthy light sources like sunlight and avoiding the dangers of blue light for the human eye. The near-ultraviolet (n-UV, ∼400 nm) LED chips are emerging in SSL technology. However, the n-UV excited color converters with high spectrum-matching degrees with n-UV chips, high efficiency and stability, and broad practical application need more research. This progress would guide future research on Ce-doped garnet phosphors and help develop new ceramic photo-convertors with tailored luminous properties.

This review article highlights the development of other cyan-emitting phosphors to fulfil the cyan gap in the emission spectrum and fabricate a white LED with high thermal and moisture stability to generate a highly efficient white light source. Developing narrow-band cyan emission will also be promising for the high color gamut displays.

## References

[B1] Ali L. Khan N. Z. Abbas M. T. Muhammad N. Alshehri S. M. Shah W. H. (2023). Synthesis and characterization of narrow band emitting phosphors for plant growth and display applications. Optik 274, 170570. 10.1016/j.ijleo.2023.170570

[B2] Amachraa M. Wang Z. Chen C. Hariyani S. Tang H. Brgoch J. (2020). Predicting thermal quenching in inorganic phosphors. Chem. Mater. 32 (14), 6256–6265. 10.1021/acs.chemmater.0c02231

[B3] Brgoch J. DenBaars S. P. Seshadri R. (2013). Proxies from *ab initio* calculations for screening efficient Ce^3+^ phosphor hosts. J. Phys. Chem. C 117, 17955–17959. 10.1021/jp405858e

[B4] Cao L. Li W. Devakumar B. Ma N. Huang X. Lee A. F. (2022). Full-spectrum white lightemitting diodes enabled by an efficient broadband green-emitting CaY_2_ZrScAl_3_O_12_: Ce^3+^ garnet phosphor. ACS Appl. Mater. Interfaces 14, 5643–5652. 10.1021/acsami.1c23286 35075892

[B5] Chan J. Cao L. Li W. Ma N. Xu Z. Huang X. (2022a). Highly efficient broad-band green emitting cerium (III)-Activated garnet phosphor allows the fabrication of blue-chip-based warm-white LED device with a superior color rendering index. Inorg. Chem. 61, 6953–6963. 10.1021/acs.inorgchem.2c00326 35476582

[B6] Chan J. Cao L. Xu Z. Huang X. (2023). Cation substitution induced highly symmetric crystal structure in cyan-green-emitting Ca_2_La_1-x_Lu_x_Hf_2_Al_3_O_12_:Ce^3+^ solid-solution phosphors with enhanced photoluminescence emission and thermal stability: Toward full-visible spectrum white LEDs. Mater. Today Phys. 35, 101130. 10.1016/j.mtphys.2023.101130

[B7] Chan J. Devakumar B. Li W. Ma N. Huang X. Lee A. F. (2022b). Full-spectrum solid-state white lighting with high color rendering index exceeding 96 based on a bright broadband green emitting phosphor. Appl. Mater. Today 27, 101439. 10.1016/j.apmt.2022.101439

[B8] Chen H. Wang Y. (2019). Sr_2_LiScB_4_O_10_:Ce^3+^/Tb^3+^: A green-emitting phosphor with high energy transfer efficiency and stability for LEDs and FEDs. Inorg. Chem. 58, 7440–7452. 10.1021/acs.inorgchem.9b00639 31074973

[B9] Denault K. A. Brgoch J. Kloß S. D. Gaultois M. W. Siewenie J. Page K. (2015). Average, and local structure, Debye temperature, and structural rigidity in some oxide compounds related to phosphor hosts. ACS Appl. Mater. Interfaces 7, 7264–7272. 10.1021/acsami.5b00445 25815799

[B10] Ding J. Wei Y. Liu W. Li Y. Wu Q. Zhou J. (2021). Highly efficient and thermally stable narrow-band cyan-emitting aluminum oxynitride phosphor for WLEDs and FEDs. Chem. Eng. J. 403, 126382. 10.1016/j.cej.2020.126382

[B11] Duke A. C. Hariyani S. Brgoch J. (2018). Ba_3_Y_2_B_6_O_15_:Ce^3+^-A high symmetry, narrow emitting blue phosphor for wide-gamut white lighting. Chem. Mater. 30, 2668–2675. 10.1021/acs.chemmater.8b00111

[B12] Fischer S. Pier T. Justel T. (2018). On the sensitization of Eu^3+^ with Ce^3+^ and Tb^3+^ by composite structured Ca_2_LuHf_2_Al_3_O_12_ garnet phosphors for blue LED excitation. Dalton Trans. 48, 315–323. 10.1039/c8dt04125c 30516763

[B13] Geng W. Zhou X. Ding J. Wang Y. (2018). NaBaY(BO_3_)_2_:Ce^3+^,Tb^3+^: A novel sharp green-emitting phosphor used for WLED and FEDs. J. Am. Ceram. Soc. 101, 4560–4571. 10.1111/jace.15693

[B14] Gong X. H. Huang J. H. Chen Y. J. Lin Y. F. Luo Z. D. Huang Y. D. (2014). Novel garnetstructure Ca_2_GdZr_2_(AlO4)_3_: Ce^3+^ phosphor and its structural tuning of optical properties. Inorg. Chem. 53 (13), 6607–6614. 10.1021/ic500153u 24915022

[B15] Guo H. Devakumar B. Li B. Huang X. (2018). Novel Na_3_Sc_2_(PO4)_3_:Ce^3+^,Tb^3+^ phosphors for white LEDs: Tunable blue-green color emission, high quantum efficiency and excellent thermal stability. Dyes Pigm 151, 81–88. 10.1016/j.dyepig.2017.12.051

[B16] Guo H. Huang X. (2018). Low-temperature solid-state synthesis and photoluminescence properties of novel high-brightness and thermal-stable Eu^3+^-activated Na_2_Lu(MoO_4_)(PO_4_) red-emitting phosphors for near-UV-excited white LEDs, J. Alloy. Comp 764, 809–814. 10.1016/j.jallcom.2018.06.156

[B17] Hakeem D. A. Pi J. W. Jung G. W. Kim S. W. Park K. (2019). Structural and photoluminescence properties of La_1-x_NaCaGa_3_PZrO_12_ doped with Ce^3+^, Eu^3+^, and Tb^3+^ . Dyes Pigments 160, 234–242. 10.1016/j.dyepig.2018.06.047

[B18] Hakeem D. A. Pi J. W. Kim S. W. Park K. (2018). New Y_2_LuCaAl_2_SiO_12_:Ln (Ln = Ce^3+^, Eu^3+^, and Tb^3+^) phosphors for white LED applications. Inorg. Chem. Front. 5, 1336–1345. 10.1039/c8qi00111a

[B19] Hua Y. Khaja Hussain S. Yu J. S. (2019). Samarium(iii) and terbium(iii) ion-doped NaLa (MoO_4_)_2_ phosphors for versatile applications. New J. Chem. 43, 10645–10657. 10.1039/c9nj01751h

[B20] Huang X. (2019a). Cyan phosphors for full-visible-spectrum lighting: Shining new light on high-CRI white pc-LEDs. Sci. Bull. 64, 1649–1651. 10.1016/j.scib.2019.09.008 36659776

[B21] Huang X. Guo H. (2018). LiCa_3_MgV_3_O_12_:Sm^3+^: A new high-efficiency white-emitting phosphor. Ceram. Int. 44, 10340–10344. 10.1016/j.ceramint.2018.03.043

[B22] Huang X. Li B. Guo H. (2017). Synthesis, photoluminescence, cathodoluminescence, and thermal properties of novel Tb^3+^-doped BiOCl green-emitting phosphors. J. Alloys Compd. 695, 2773–2780. 10.1016/j.jallcom.2016.11.224

[B23] Huang X. (2019b). New red phosphors enable white LEDs to show both high luminous efficacy and color rendering index. Sci. Bull. 64, 879–880. 10.1016/j.scib.2019.06.003 36659749

[B24] Jaffe H. W. (1951). The role of yttrium and other minor elements in the garnet group 1. Am. Mineral. 36, 133–155.

[B25] Jia L. Devakumar B. Sun L. Wang S. Sun Q. Huang X. (2020). Full-visible-spectrum lighting enabled by an excellent cyan-emitting garnet phosphor. J. Mater. Chem. C 8, 4934–4943. 10.1039/d0tc00006j

[B26] Jiao M. Dong L. Xu Q. Zhang L. Wang D. Yang C. (2020). The structures and luminescence properties of Sr_4_Gd_3_Na_3_(PO_4_)_6_F_2_:Ce^3+^, Tb^3+^ green phosphors with zero-thermal quenching of Tb^3+^ for WLEDs. Dalton Trans. 49, 667–674. 10.1039/c9dt04320a 31844868

[B27] Katayama Y. Viana B. Gourier D. Xu J. Tanabe S. (2016). Photostimulation induced persistent luminescence in Y_3_Al_2_Ga_3_O_12_:Cr^3+^ . Opt. Mater Express 6, 1405. 10.1364/ome.6.001405

[B28] Khan N. Z. Khan S. A. Jalil A. Wang F. Mehmood I. Abbas M. T. (2022). Structural development and luminescent enhancement of CaAlSiN_3_: Eu^2+^ phosphor via replacing Al^3+^ by Ga^3+^ . J. Alloys Compd. 897, 162485. 10.1016/j.jallcom.2021.162485

[B29] Khan N. Z. Khan S. A. Zhan L. Jalil A. Ahmed J. Khan M. M. (2021). Synthesis, structure and photoluminescence properties of Ca_2_YTaO_6_: Bi^3+^\Eu^3+^ double perovskite white light emitting phosphors. J. Alloys Compd. 868, 159257. 10.1016/j.jallcom.2021.159257

[B30] Khan S. A. Khan N. Z. Muhammad N. Lin F. Runowski M. Ahmed J. (2023a). Highly efficient and tunable broadband UV excitable Ba_9_Lu_2_Si_6_O_24_: Eu^2+^, Mn^2+^ single-phase white-light-emitting phosphors. J. Alloys Compd. 938, 168650. 10.1016/j.jallcom.2022.168650

[B31] Khan S. A. Khan N. Z. Sohail M. Runowski M. Xu X. Agathopoulos S. (2023b). Recent developments of lead-free halide-perovskite nanocrystals: Synthesis strategies, stability, challenges, and potential in optoelectronic applications. Mater. Today Phys. 34, 101079. 10.1016/j.mtphys.2023.101079

[B32] Kim D. Bae J. S. Hong T. E. Hui K. N. Kim S. Kim C. H. (2016). Color-tunable, and highly luminous N_3_−-doped Ba_2-x_Ca_x_SiO_4-δ_N_2/3δ_: Eu^2+^ (0.0≤x≤1.0) phosphors for white NUV-led. ACS Appl. Mater. Interfaces 8, 17371–17381. 10.1021/acsami.6b02778 27322133

[B33] Kireev P. S. Samokhvalov M. (1978). Semiconductor physics. Moscow: Mir Publishers.

[B34] Leaño J. L. Lazarowska A. Mahlik S. Grinberg M. Sheu H.-S. Liu R.-S. (2018). Disentangling red emission and compensatory defects in Sr[LiAl_3_N_4_]:Ce^3+^ phosphor. Chem. Mater. 30, 4493–4497. 10.1021/acs.chemmater.8b01561

[B35] Lee H. S. Yoo J. W. (2011). Yellow phosphors coated with TiO_2_ for the enhancement of photoluminescence and thermal stability. Appl. Surf. Sci. 257, 8355–8359. 10.1016/j.apsusc.2011.03.137

[B36] Li B. Huang X. Guo H. Zeng Y. (2018). Energy transfer and tunable photoluminescence of LaBWO_6_:Tb^3+^, Eu^3+^ phosphors for near-UV white LEDs. Dyes Pigm 150, 67–72. 10.1016/j.dyepig.2017.11.003

[B37] Li J. Li J.-G. Liu S. Li X. Sun X. Sakka Y. (2016). The development of Ce^3+^-activated (Gd,Lu)_3_Al_5_O_12_ garnet solid solutions as efficient yellow-emitting phosphors. Sci. Technol. Adv. Mater 14, 054201. 10.1088/1468-6996/14/5/054201 PMC5090367 27877604

[B38] Li W. Ma N. Devakumar B. Huang X. (2022). Blue-light-excitable broadband yellow emitting CaGd_2_HfSc(AlO_4_)_3_:Ce^3+^ garnet phosphors for white light-emitting diode devices with improved color rendering index. Mater. Today Chem. 23, 100638. 10.1016/j.mtchem.2021.100638

[B39] Li W. Qiu M. Li Y. Zhang S. Li Q. Lin W. (2020). Energy transfer and multicolor-tunable emissions of Sr_3_La_6_(SiO_4_)_6_:Ce^3+^,Tb^3+^,Eu^3+^ . J. Elect. Mater. 49, 1404–1411. 10.1007/s11664-019-07813-3

[B40] Li Y. Q. Hirosaki N. Xie R. J. Takeda T. Mitomo M. (2008). Yellow-Orange-emitting CaAlSiN_3_:Ce^3+^ phosphor: Structure, photoluminescence, and application in white LEDs. Chem. Mater. 20, 6704–6714. 10.1021/cm801669x

[B41] Li Y. Shao Y. Zhang W. Ye S. Zhou J. Chen M. (2021). Bismuth activated, narrow-band, cyan garnet phosphor Ca_3_Y_2_Ge_3_O_12_:Bi^3+^ for near ultraviolet-pumped white LED application. J. Am. Ceram. Soc. 104, 6299–6308. 10.1111/jace.18015

[B42] Li Z. Li S. Xin S. Bian Q. He M. Ge Zhu A. (2023). A nitriding garnet structure cyan emitting phosphor Ca_2_(Y, Ce)Hf_2_(Al, Si)_3_(O, N)_12_ with high efficiency and excellent thermal stability. J. Alloys Compd. 944, 169253. 10.1016/j.jallcom.2023.169253

[B43] Liang J. Devakumar B. Sun L. L. Wang S. Y. Sun Q. Huang X. Y. (2020a). Full-visiblespectrum lighting enabled by an excellent cyan-emitting garnet phosphor. J. Mater. Chem. C 8, 4934–4943. 10.1039/d0tc00006j

[B44] Liang J. Sun L. L. Wang S. Y. Sun Q. Devakumar B. Huang X. Y. (2020b). Filling the cyan gap toward full-visible-spectrum LED lighting with Ca_2_LaHf_2_Al_3_O_12_: Ce^3+^ broadband green phosphor. J. Alloy Compd. 836 (25), 155469. 1–155469.7. 10.1016/j.jallcom.2020.155469

[B45] Lin C. C. Tsai Y. T. Johnston H. E. Fang M. H. Yu F. J. Zhou W. Z. (2017). Enhanced photoluminescence emission and thermal stability from introduced cation disorder in phosphors. J. Am. Chem. Soc. 139, 11766–11770. 10.1021/jacs.7b04338 28764327

[B46] Liu C. Y. Xia Z. G. Molokeev M. S. Liu Q. L. (2015). Synthesis, crystal structure, and enhanced luminescence of garnet-type Ca_3_Ga_2_Ge_3_O_12_:Cr^3+^ by co-doping Bi^3+^ . J. Am. Ceram. Soc. 98, 1870–1876. 10.1111/jace.13553

[B47] Luo Y. Xia Z. (2014). Effect of Al/Ga substitution on photoluminescence and phosphorescence properties of garnet-type Y_3_Sc_2_Ga_3–x_Al_x_O_12_:Ce^3+^ phosphor. J. Phys. Chem. C 118, 23297–23305. 10.1021/jp507695n

[B48] Ma N. Li W. Devakumar B. Zhang Z. Huang X. (2021). Utilizing energy transfer strategy to produce efficient green luminescence in Ca_2_LuHf_2_Al_3_O_12_:Ce^3+^, Tb^3+^ garnet phosphors for high quality near-UV-pumped warm-white LEDs. J. Colloid Interface Sci. 601, 365–377. 10.1016/j.jcis.2021.05.108 34087597

[B49] Malysa B. Meijerink A. Jüstel T. (2018). Temperature dependent Cr^3+^ photoluminescence in garnets of the type X_3_Sc_2_Ga_3_O_12_ (X = Lu, Y, Gd, La). J. Lumin 202, 523–531. 10.1016/j.jlumin.2018.05.076

[B50] Park J. Lee S. J. Kim Y. J. (2013). Evolution of luminescence of Sr_2-y-z_CazSi(O_1-x_N_x_)_4_: yEu^2+^ with N^3−^, Eu^2+^, and Ca^2+^ substitutions. Cryst. Growth Des. 13, 5204–5210. 10.1021/cg400751n

[B51] Pasinski D. Zych E. Sokolnicki J. (2016). The effect of N^3-^ substitution for O^2-^ on optical properties of YAG: Ce^3+^ phosphor. J. Alloy Compd. 668, 194–199. 10.1016/j.jallcom.2016.01.223

[B52] Qiang Y. Pan Z. Ye X. Liang M. Xu J. Huang J. (2018). Ce^3+^ doped BaLu_2_Al_4_SiO_12_: A promising green-emitting phosphor for white LEDs. J. Luminescence 203, 609–615. 10.1016/j.jlumin.2018.07.002

[B53] Setlur A. A. Heward W. J. Gao Y. Srivastava A. M. Chandran R. G. Shankar M. V. (2006). Crystal chemistry and luminescence of Ce^3+^-doped Lu_2_CaMg_2_(Si, Ge)_3_O_12_ and its use in LED based lighting. Chem. Mater. 18, 3314–3322. 10.1021/cm060898c

[B54] Skruodiene M. Katelnikovas A. Vasylechko L. Skaudzius R. (2019). Tb^3+^ to Cr^3+^ energy transfer in a co-doped Y_3_Al_5_O_12_ host. J. Lumin 208, 327–333. 10.1016/j.jlumin.2018.12.048

[B55] Skruodiene M. Misevicius M. Sakalauskaite M. Katelnikovas A. Skaudzius R. (2016). Doping effect of Tb^3+^ ions on luminescence properties of Y_3_Al_5_O_12_:Cr^3+^ phosphor. J. Lumin 179, 355–360. 10.1016/j.jlumin.2016.07.041

[B56] Strobel P. de Boer T. Weiler V. Schmidt P. J. Moewes A. Schnick W. (2018). Luminescence of an oxonitridoberyllate: A study of narrowband cyan emitting Sr[Be_6_ON_4_]:Eu^2+^ . Chem. Mater. 30, 3122–3130. 10.1021/acs.chemmater.8b01256

[B57] Sun L. L. Devakumar B. Liang J. Wang S. Y. Sun Q. Huang X. Y. (2020a). A broadband cyan-emitting Ca_2_LuZr_2_(AlO_4_)_3_: Ce^3+^ garnet phosphor for near-ultraviolet-pumped warm-white light-emitting diodes with an improved color rendering index. J. Mater. Chem. C 8, 1095–1103. 10.1039/c9tc04952e

[B58] Sun Q. Wang S. Y. Sun L. L. Liang J. Devakumar B. Huang X. Y. (2020b). Achieving full-visible-spectrum LED lighting via employing an efficient Ce^3+^-activated cyan phosphor. Mater. Today. Energy 17, 100448. 10.1016/j.mtener.2020.100448

[B59] Sun X. Zhang C. Wu J. Zhu P. Zhang X. Hang Y. (2016). A novel blue-emitting KCa_4_(BO_3_)_3_:Ce^3+^ phosphor for white LED application. J. Rare Earth. 34, 571–575. 10.1016/s1002-0721(16)60063-7

[B60] Tolhurst T. M. Strobel P. Schmidt P. J. Schnick W. Moewes A. (2017). Direct measurements of energy levels and correlation with thermal quenching behavior in nitride phosphors. Chem. Mater. 29 (18), 7976–7983. 10.1021/acs.chemmater.7b02974

[B61] Vijayakumar M. Uma V. Arunkumar S. Marimuthu K. (2019). Spectroscopic studies on Sm^3+^:Tb^3+^ codoped aluminium borotellurite glasses for white light applications. AIP Conf. Proc. 2115, 030244. 10.1063/1.5113083

[B62] Vijayakumar R. Devakumar B. Huang X. (2021). Energy transfer induced colortunable emissions from Ba_2_Gd_5_B_5_O_17_:Ce^3+^/Tb^3+^ borate phosphors for white LEDs. J. Lumin. 229, 117685. 10.1016/j.jlumin.2020.117685

[B63] Viswanath N. S. M. Krishnamurthy Grandhi G. Choi H. Min Kim S. (2021). Ha tran huu, hyuk choi, ha jun Kim, seong min Kim, hyun you Kim, chan-jin park, won bin im, zero-thermal-quenching and improved chemical stability of a UCr_4_C_4_-type phosphor via crystal site engineering. Chem. Eng. J. 420, 127664. 10.1016/j.cej.2020.127664

[B64] Wang W. Tao M. Liu Y. Wei Y. i. Xing G. Dang P. (2019). Photoluminescence control of UCr_4_C_4_ -type phosphors with superior luminous efficiency and high color purity via controlling site selection of Eu^2+^ activators. Chem. Mater. 31 (21), 9200–9210. 10.1021/acs.chemmater.9b04089

[B65] Wang X. C. Zhao Z. Y. Wu Q. S. Li Y. Y. Wang Y. H. (2016). Synthesis, structure and photoluminescence properties of Ca_2_LuHf_2_(AlO_4_)_3_:Ce^3+^, a novel garnet-based cyan light-emitting phosphor. J. Mater Chem. C 4 (48), 11396–11403. 10.1039/c6tc03933b

[B66] Wang X. C. Wang Y. H. (2015). Synthesis, structure, and photoluminescence properties of Ce^3+^-doped Ca_2_YZr_2_Al_3_O_12_: A novel garnet phosphor for white LEDs. J. Phys. Chem. C 119 (28), 16208–16214. 10.1021/acs.jpcc.5b01552

[B67] Wang Z. Meng F. Feng Q. Shi S. Qiu L. Sun G. (2022). Efficient green quasi -Two-Dimensional perovskite light-emitting diodes based on mix-interlayer. Front. Chem. 9, 825822. 10.3389/fchem.2021.825822 35111732 PMC8802909

[B68] Wu D. Liu L. Duan H. Wang J. Zou W. Peng J. (2022). A comparison research on replacements of Ba^2+^ by Lu^3+^ and Ba^2+^-Si^4+^ by Lu^3+^-Al^3+^ in BaSi_2_O_2_N_2_:Eu phosphors. J. Rare Earths 40, 20–28. 10.1016/j.jre.2021.07.006

[B69] Wu H. Sun Z. Gan S. Li L. (2019). Effects of alkali metal as charge compensator on the luminescence properties of ZnWO_4_:Eu^3+^ phosphors synthesized by solid-state reaction. J. Photochem. Photobiol. A 368, 258–262. 10.1016/j.jphotochem.2018.09.048

[B70] Wu J. P. Zhuang W. D. Liu R. H. Liu Y. H. Gao T. Y. Yan C. P. (2021). Broadband near-infrared luminescence and energy transfer of Cr^3+^, Ce^3+^ co-doped Ca_2_LuHf_2_Al_3_O_12_ phosphors. J. Rare Earths 39, 269–276. 10.1016/j.jre.2020.05.008

[B71] Xiao Y. Hao Z. Zhang L. Xiao W. Wu D. Zhang X. (2017). Highly efficient green-emitting phosphors Ba_2_Y_5_B_5_O_17_ with low thermal quenching due to fast energy transfer from Ce^3+^ to Tb^3+^ . Inorg. Chem. 56, 4538–4544. 10.1021/acs.inorgchem.7b00085 28358516

[B72] Xu J. Ueda J. Tanabe S. (2017). Toward tunable and bright deep-red persistent luminescence of Cr^3+^ in garnets. J. Am. Ceram. Soc. 100, 4033–4044. 10.1111/jace.14942

[B73] Xu Z. Devakumar B. Ma N. Li W. Huang X. (2022). High-brightness cyan-emitting Eu^2+^- activated orthophosphate phosphors for near-UV-pumped white LEDs. J. Lumin. 243, 118640. 10.1016/j.jlumin.2021.118640

[B74] Yan C. Liu Z. Zhuang W. Liu R. Xing X. Liu Y. (2017). YScSi_4_N_6_C:Ce^3+^ A broad cyan-emitting phosphor to weaken the cyan cavity in full spectrum white light-emitting diodes. Inorg. Chem. 56, 11087–11095. 10.1021/acs.inorgchem.7b01408 28841298

[B75] Yan J. Zhang Z. Milic´evic´ B. Li J. Liang Q. Zhou J. (2019). The enhancement of emission intensity and enlargement of color gamut by a simple local structure substitution with highly thermal stability preserved. Opt. Mater. 95, 109201. 10.1016/j.optmat.2019.109201

[B76] Yang C. Peng Z. J. Hu J. Zhao P. H. Shen S. H. Song K. X. (2022). Nitriding improvement of luminescence properties and energy-transfer behaviors of LaMgAl_1-x_Si_3_x/_4_O_19_- 3x/2nx: 0.55Ce^3+^ 0.25 Tb^3+^ phosphors for UV-light pumping lamps. Opt. Mater. 124, 111980. 10.1016/j.optmat.2022.111980

[B77] Yang L. Wan Y. Weng H. Huang Y. Chen C. Seo H. J. (2016). Luminescence, and color center distributions in K_3_YB_6_O_12_:Ce^3+^ phosphor. J. Phys. D. Appl. Phys. 49, 325303. 10.1088/0022-3727/49/32/325303

[B78] Yoder H. S. Keith M. L. (1951). Complete substitution of aluminum for silicon-the system-3MnO•Al_2_O_3_•3SiO_2_-3Y_2_O_3_•5Al_2_O_3_ . Am. Mineral. 36, 519–533.

[B79] You S. H. Li S. X. Wang L. Takeda T. Hirosaki N. Xie R. J. (2021). Ternary solid solution phosphors Ca_1-x-y_LixAl_1-x-y_Si_1+x+y_N_3-y_O_y_: Ce^3+^ with enhanced thermal stability for high-power laser lighting. Chem. Eng. J. 404 (15), 126575. 10.1016/j.cej.2020.126575

[B80] Yuan H. Massuyeau F. Gautier N. Kama A. Faulques E. Shen Q. (2019). Doped lead halide white phosphors for very high efficiency and ultra-high color rendering. Angew. Chem. Int. Ed. 59, 2802–2807. 10.1002/anie.201910180 31830354

[B81] Zhang L. L. Zhang S. Hao Z. D. Zhang X. Pan G. H. Luo Y. S. (2018). A high efficiency broad-band near-infrared Ca_2_LuZr_2_Al_3_O_12_:Cr^3+^ garnet phosphor for blue LED chips. J. Mater Chem. C 6, 4967–4976. 10.1039/c8tc01216d

[B82] Zhang Q. Li J. Jiang W. Lin L. Ding J. Brik M. G. (2021a). CaY_2_Al_4_SiO_12_:Ce^3+^, Mn^2+^: A single component phosphor to produce high color rendering index WLEDs with a blue chip. J. Mater. Chem. C 9, 11292–11298. 10.1039/d1tc01770e

[B83] Zhang T. He S.-J. Wang D.-K. Jiang N. Lu Z.-H. (2016). A multi-zoned white organic light-emitting diode with high CRI and low color temperature. Sci. Rep. 6, 20517. 10.1038/srep20517 26842934 PMC4740810

[B84] Zhang X. Zhang D. Kan D. Wu T. Song Y. Zheng K. (2020). Crystal structure, luminescence properties and application performance of color tuning Y_2_Mg_2_Al_2_Si_2_O_12_:Ce^3+^, Mn^2+^ phosphors for warm white light emitting diodes. Mater. Adv. 1, 2261–2270. 10.1039/d0ma00556h

[B85] Zhang Z. J. Devakumar B. Wang S. Y. Sun L. L. Ma N. Li W. (2021b). Using an excellent near-UV-excited cyan-emitting phosphor for enhancing the color rendering index of warm-white LEDs by filling the cyan gap. Mater. Today. Chem. 20, 100471. 10.1016/j.mtchem.2021.100471

[B86] Zhao J. Guo C. Li T. Su X. Zhang N. Chen J. (2016). Synthesis, electronic structure and photoluminescence properties of Ba2BiV3O11: Eu3+ red phosphor. Dyes Pigm 132, 159–166. 10.1016/j.dyepig.2016.04.052

[B87] Zheng Y. L. Zhuang W. D. Xu H. B. Chen M. Y. Li Y. F. Xing X. R. (2019). Polyhedral distortion control of unusual photoluminescence color tuning in garnet phosphors via cation substitution. J. Am. Ceram. Soc. 2, 2593. 10.1111/jace.16109

[B88] Zhou W. Pan F. Zhou L. Hou D. Huang Y. Tao Y. (2016). Site occupancies, luminescence, and thermometric properties of LiY_9_(SiO_4_)_6_O_2_:Ce^3+^ phosphors. Inorg. Chem. 55, 10415–10424. 10.1021/acs.inorgchem.6b01656 27700068

[B89] Zhou Y. Zhuang W. Hu Y. Liu R. Jiang Z. Liu Y. (2017). A broad-band orange-yellow-emitting Lu_2_Mg_2_Al_2_Si_2_O_12_:Ce^3+^ phosphor for application in warm white light-emitting diodes. RSC Adv. 7, 46713–46720. 10.1039/c7ra08760h

[B90] Zhu Y. L. Liang Y. J. Liu S. Q. Li H. R. Chen J. H. Lei W. (2018). Design of hierarchical composite silicate for full-color and high thermal stability phosphors. Chem. Eng. J. 345, 327–336. 10.1016/j.cej.2018.03.182

[B91] Zhuang J. Q. Xia Z. G. Liu H. K. Zhang Z. P. Liao L. B. (2011). The improvement of moisture resistance and thermal stability of Ca_3_SiO_4_Cl_2_: Eu^2+^ phosphor coated with SiO_2_ . Appl. Surf. Sci. 257, 4350–4353. 10.1016/j.apsusc.2010.12.055

[B92] Zhuo Y. Tehrani A. M. Oliynyk A. O. Duke A. C. Brgoch J. (2018). Identifying an efficient, thermally robust inorganic phosphor host via machine learning. Nat. Commun. 9, 4377. 10.1038/s41467-018-06625-z 30348949 PMC6197245

